# ApoE3 Christchurch and tau interaction as a protective mechanism against Alzheimer's disease

**DOI:** 10.1002/alz.70396

**Published:** 2025-07-10

**Authors:** Paula Perez‐Corredor, Said Arevalo‐Alquichire, Randall C. Mazzarino, Michael O'Hare, Andres F. Muriel‐Torres, Guido N. Vacano, Timothy E. Vanderleest, William P. Miller, Lina Pineda‐Lopez, Shivani Patel, Robert A. Obar, Nihat Polat, Leo A. Kim, Joseph F. Arboleda‐Velasquez, Claudia Marino

**Affiliations:** ^1^ Schepens Eye Research Institute of Mass Eye and Ear and Department of Ophthalmology Harvard Medical School Boston Massachusetts USA; ^2^ Wellcome‐Wolfson Institute for Experimental Medicine Queen's University Belfast Belfast UK; ^3^ Vacano Informatics LLC Arvada Colorado USA; ^4^ Department of Cell Biology Harvard Medical School Boston Massachusetts USA; ^5^ Department of Ophthalmology School of Medicine Inonu University Malatya Turkey; ^6^ Sealy Institute for Drug Discovery The University of Texas Medical Branch at Galveston Galveston Texas USA; ^7^ Mitchell Center for Neurodegenerative Diseases Department of Neurology University of Texas Medical Branch at Galveston Galveston Texas USA

**Keywords:** Alzheimer's disease, ApoE3 Christchurch, apolipoprotein E, Dkk1, oligomerization, tau

## Abstract

**INTRODUCTION:**

We described a protected case with familial Alzheimer's disease, homozygous for apolipoprotein E3 (APOE3) Christchurch variant (ApoE3Ch), exhibiting low tau protein levels despite genetic predisposition to the disease due to presenilin (PSEN)1‐E280A. We reported the loss of interaction between ApoE3Ch and heparan sulfate proteoglycans (HSPGs) as a critical protective pathway. Here, we characterized differential interacting partners for both wild‐type and Christchurch variants to identify additional protective mechanisms of ApoE3Ch.

**METHODS:**

We performed pull‐down of mouse brain lysates using His‐tag‐ApoE3 recombinant proteins and determined interacting partners of ApoE3 via mass‐spectrometry. We then performed in vitro and in vivo assays to validate the top interactors.

**RESULTS:**

We found enhanced binding of ApoE3Ch to tau and Dickkopf‐1 (Dkk1, a WNT/β‐catenin antagonist) that resulted in reduced tau aggregation in vitro. We demonstrated that ApoE3Ch interacts directly with Dkk1 and tau, reducing tau pathology. These findings supported the hypothesis of novel protective effects of direct ApoE3Ch interactions.

**Highlights:**

- ⁠Apolipoprotein E3 (ApoE3) Christchurch variant (ApoE3Ch) exhibits different protein interaction profiles compared to wild‐type ApoE3, as revealed by proteomic analyses and pull‐down experiments.
- The ApoE3Ch variant alters the protein's interaction with tau, thus affecting its aggregation in a tau biosensor cell assay and the retina of microtubule‐associated protein tau (MAPT*P301S) transgenic mice.
- ⁠Gene ontology and pathway analyses indicate that ApoE3Ch interactors are associated with brain‐related disorders and specific upstream regulators, including MAPT, a gene encoding for tau.
- ⁠Protein–protein interaction studies showed increased binding of ApoE3Ch to Dickkopf1 (Dkk1), a Wnt/β‐catenin pathway antagonist, as compared to ApoE3WT, thus indicating that multiple protective mechanisms are regulated by the ApoE3Ch variant
- Our study uncovers a novel protective effect of the ApoE3Ch variant against tau pathology, thus proposing new insights into Alzheimer's disease mechanisms and potential therapeutic targets

## 1 BACKGROUND

Alzheimer's disease is the most common form of dementia worldwide.^1^ Autosomal dominant Alzheimer's disease (ADAD) is a form of AD caused by genetic mutations affecting the *APP* (amyloid precursor protein) gene or genes encoding enzymes involved in APP processing, such as the presenilin 1 (*PSEN1)* and presenilin 2 (*PSEN2)*.^2^ Specifically, an aggressive form of ADAD is caused by the *PSEN1* E280A variant due to the substitution of glutamic acid for alanine at codon 280 of this gene. Individuals with this disease typically have a median age of onset of cognitive decline at 44 years of age.^3^ The Paisa kindred in Colombia is the largest known family with ADAD due to the PSEN1 E280A variant.^4^ Among these families, we identified a woman who remained cognitively unimpaired for about three decades beyond the expected age of mild cognitive impairment (MCI) onset for *PSEN1* E280A mutation carriers, despite high Pittsburgh compound B positron emission tomography (PET) measurements of Aβ burden.^5^ Conversely, both tau burden and hippocampal atrophy were comparable to young carriers with typical MCI from the same kindred. PET measurements of the cerebral metabolic rate for glucose (CMRgl) indicated the presence of high glucose metabolism in brain regions highly impacted by AD, supporting the conclusion that the brain of this individual was functionally preserved. Genetic analyses revealed homozygosity for a rare variant in the apolipoprotein E (*APOE)* gene, referred to as *APOE3* Christchurch variant (Ch), substituting arginine for serine at position 136 based on “classic” nomenclature, equivalent to codon position 154 according to current nomenclature.^5^

Apolipoprotein E is characterized by different variants with neutral (ApoE3), protective (ApoE2), or detrimental (ApoE4) roles in Alzheimer's disease pathogenesis.^6^ ApoE variants differ from the relatively neutral variant ApoE3 (referred to as ApoE3WT) with either the presence of an R112 residue, characteristic of ApoE4, or a C158, characteristic of ApoE2, according to the classic nomenclature.^7^ Previous studies have shown that differences in the binding affinity of these ApoE variants to heparan sulfate proteoglycans (HSPGs) might have a pathophysiological role by bringing lipid particles in proximity to cell receptors.^8^, ^9^ Interestingly, like ApoE2, ApoE3Ch shows reduced binding to HSPGs. Thus, we hypothesized that the reduced interaction between ApoE and HSPGs due to the Ch variant (located in one of the two HSPG‐binding regions) could be the protective mechanism against ADAD.^5^

It has been proposed by others that astrocyte‐derived ApoE has functions other than lipid transport that are critical for brain development and synaptic plasticity^10^; therefore, we cannot exclude the possibility that the Ch variant may facilitate other protective mechanisms via HSPG‐independent biochemical pathways. Further, whether the Christchurch variant favors conformational changes in ApoE3 that lead to a loss of HSPG‐ApoE receptor interactions and a loss or gain of interaction(s) with other biochemical pathways remains unknown. Thus, using biomolecular, proteomic, and in vivo approaches, we compared the interacting partners of ApoE3 and ApoE3Ch variants in mouse brain to determine unique interactors of ApoE3Ch that might confer protection against ADAD.

Overall, our findings demonstrate direct interactions between the protective Christchurch variant and proteins that are involved in AD pathogenesis, supporting the hypothesis of HSPG‐independent novel protective pathways.

## 2 METHODS

### 2.1 Protein expression and purification

Recombinant His‐tagged ApoE3 wild‐type (ApoE3 WT) and ApoE3Ch proteins were produced either in *E. coli* (Innovagen) or in human embryonic kidney (HEK293 or Expi293F) cells, as previously reported.^11^ For the latter proteins, cDNAs for APOE3 with and without the Christchurch variant were obtained as synthetic DNA gene fragments (gBlocks, Integrated DNA Technologies) with His‐tags at the C‐terminal. gBlocks were Gibson ligated into a linear pcDNA3 plasmid.^12^ Primers used for successful insertion: reverse, 5′‐GGTGAAGCTTAAGTTTAAACG‐3′; and forward, 5′‐TGAGGATCCACTAGTCCAGTG‐3′. Correct insertion was verified by DNA sequencing (Quintara Biosciences). Expi293F cells were transiently transfected to express the APOE3 constructs using an ExpiFectamine293 Transfection Kit according to the manufacturer's protocol (Thermo Fisher Scientific, Waltham, MA, cat. A14635). Three days post‐transfection, the conditioned media were harvested and concentrated by centrifugation and sterilized using a 0.2 µm PES filter. Filtered supernatants were then loaded onto a His‐Trap column (Cytiva) attached to an NGC medium‐pressure liquid chromatography system (BioRad), washed with Ni‐NTA Loading Buffer (20 mM sodium phosphate, 500 mM sodium chloride, 20 mM imidazole, pH 7.4, cat. S1423S) and then eluted with a gradient of increasing ionic strength using Ni‐NTA Elution Buffer (20 mM sodium phosphate, 500 mM sodium chloride, 500 mM imidazole, pH 7.4. cat. S1423S). Recombinant tau monomers (tau‐441, rPeptide, Watkinsville, GA cat. T‐1001‐2) or preformed tau fibrils (rPeptide, Watkinsville, GA, cat. TF‐1001‐2) were obtained from rPeptide.

### 2.2 Mouse brain lysates

Three‐month old C57BL/6J female mice (*n* = 3, Jackson Laboratory) were housed in regular diet and light/dark cycle following the Schepens Eye Research Institute Institutional Animal Care and Use Committee‐approved protocol. Frontal cortices were dissected upon induced euthanasia of the mice with saturating CO_2_ gas, followed by cervical dislocation. The frontal cortex was isolated and homogenized using a mechanical homogenizer tip (three times, 15″; PowerGen 125), strained with 70 mm cell strainer and centrifuged twice at 12,000 rpm for 30 min at 4°C. To obtain brain homogenates, we tested three different lysis buffers to obtain the optimal protein yield for the protein pull‐down with His‐tag selective nickel beads. In short, mouse brain lysates (100 µg) were prepared in: Buffer 1 (100 mM, 10% glycerol, 20 mM Tris‐HCl (pH = 8), 5 mM MgCl_2_, 0.1% Tween 20), Buffer 2 (50 mM Tris‐HCl (pH = 8), 150 mM NaCl, 2 mM ethylenediaminetetraactic acid [EDTA], 1% sodium dodecyl sulfate [SDS], 0.5% Triton X‐100), or Buffer 3 (20 mM sodium phosphate, 300 mM NaCl, 10 mM Imidazole) and incubated with ApoE3 WT or ApoE3Ch (0.05 µg/µL) for 5 h at 4°C and subsequently incubated with 40 µL of NEBExpress Ni‐NTA magnetic beads (New England Biolabs, Ipswich, MA, cat. S1423S) for 30 min (Figure S1A). Proteins were eluted using imidazole. For validation, we ran a native gel (10% Mini‐PROTEAN TGX Stain‐Free gel, Biorad, Hercules, CA, cat. 4568031), stained with Coomassie blue (Thermo Fisher Scientific, Waltham, MA, cat. 20279) and analyzed the pulled‐down interactors via mass spectrometry (Figures S1B, C, S2). Sodium dodecyl sulfate‐polyacrylamide gel electrophoresis (SDS‐PAGE) revealed that “buffer 3” retained the highest number of interactors and lacked compounds that might affect native interactions, because of the high interactor yield in that buffer. This buffer was prepared from the NEBExpress Ni‐NTA Magnetic beads kit (IMAC buffer, 2 M imidazole and H_2_O, cat. S1423S). We added phosphatase and protease inhibitors (Complete EDTA‐free protease inhibitor, NC1921673, PhosSTOP, Millipore Sigma, Burlington, MA, cat. 4906837001). We further validated the pull‐down using silver staining and Western blotting of His‐Tag and ApoE (Figure S1D).

**RESEARCH IN CONTEXT**

1. **Systematic review**: We searched the Medline and PubMed databases for articles on the interacting partners of apolipoprotein E3 (ApoE3) in the context of Alzheimer's disease (AD). Other approaches have identified the interaction between ApoE3 and ApoE4 variants with tau and amyloid beta. However, we found no previous attempts studying the protective variant ApoE3Ch, which provides extreme protection against autosomal dominant AD (ADAD).
2. **Interpretation**: We studied the interactome between ApoE3 wild‐type (WT) and Christchurch variant (ApoE3Ch) via mass spectrometry. We identify that ApoE3Ch differentially interacts with proteins related to neurodegenerative diseases, such as tau and DKK1, reducing the pathological effects in AD models.
3. **Future directions**: Our manuscript proposes alternative mechanisms of protection behind the ApoE3Ch variant that will inform the future development of therapies against AD.

### 2.3 Immunoprecipitation assays

Recombinant ApoE3 variants derived from both *E. coli* and HEK293 cells were incubated at a 0.05 µg/µL concentration with mouse brain tissue homogenate obtained from *n* = 3 C57BL/6J female mice (Jackson Lab.) for 5 h in a final volume of 300 µL. Proteins were immunoprecipitated (IP) using nickel beads (New England Biolabs, cat. S1423S) according to the manufacturer's instructions. Briefly, samples were incubated with bead slurry for 30 min and eluted in elution buffer (20 mM sodium phosphate, 300 mM NaCl, 500 mM imidazole) after washing the beads three times with wash buffer (20 mM sodium phosphate, 300 mM NaCl, and 20 mM imidazole). As loading control, 10% of the sample was used for input controls and fractionated by SDS‐PAGE. Proteins were subsequently analyzed either via Western blotting or mass spectrometry.

Recombinant human Dkk1 protein (R&D Systems, Minneapolis, MN, cat. 11495‐DK‐100/CF) and recombinant ApoE3 variants (ApoE3WT and ApoE3Ch) were co‐IP using Ni‐NTA (New England Biolabs, cat. S1423L) according to the manufacturer's protocol with minor changes. Proteins were added at target concentrations in binding buffer, and 40 µL of sample was removed and frozen to save as input. 40 µL of Ni‐NTA magnetic beads were added to each reaction and allowed to interact overnight in a rotator at 4°C. Samples were washed thrice in chilled IMAC wash buffer with 40 mM imidazole. Samples were eluted in 80 µL IMAC elution buffer, then diluted with 4X Laemmli reducing buffer (Boston Bioproducts, Milford, MA cat. BP‐110R), boiled for 5 min, then stored at −80°C until further analysis. Samples were analyzed via Western blotting as reported below.

### 2.4 Western blotting

IP fractions were analyzed via Western blotting under native or denaturing conditions in 1x Laemmli buffer (Boston Bioproducts) using 10% Mini‐PROTEAN TGX Stain‐Free gels (Bio‐Rad) run at 90 V. Proteins were subsequently transferred onto a polyvinylidene fluoride (PVDF) membrane using a dry‐transfer system (iBlot, Thermo Fisher Scientific). Membranes were blocked for 1 h using Odyssey blocking buffer (LI‐COR) and probed with primary antibodies: anti‐6X His tag antibody (1:2000, Abcam, cat. ab9108); anti‐Apolipoprotein E (1:1000 or 1:500, Millipore Sigma, cat. 178479); anti‐tau antibody (clone TAU‐5; 1:1000 Abcam cat. ab80579); anti‐Dkk1 (1:1000 Thermo Fisher Scientific, cat. MA5‐32229); anti‐phospho‐tau (Ser396) (1:1000, Thermo Fisher Scientific, cat. 44‐752G). Membranes were washed three times in Tris‐buffered saline tween‐20 (TBS‐T), then incubated with either LI‐COR donkey anti‐rabbit or goat anti‐mouse secondary antibodies (1:10,000) for 1 h at room temperature. Membranes were washed an additional three times before detection using either a Licor Odyssey image analyzer or an iBright FL1500 (Thermo Fisher Scientific).

### 2.5 Silver staining

IP proteins were evaluated with silver staining using the Pierce Silver stain kit (Thermo Fisher Scientific, cat. 24612) according to the manufacturer's instructions. Briefly, proteins were separated using SDS‐PAGE, and the gel was washed twice with ultrapure water for 5 min. The gel was then fixed with a 30% ethanol:10% acetic acid solution for 15 min and washed twice with a 10% ethanol solution for 5 min. The gel was then rinsed once with ultrapure water, incubated for 30 min in the stain working solution, and washed twice in ultrapure water for 20 s. Development was quenched by incubating the gel twice for 10 min in a 5% acetic acid solution.

### 2.6 Interactome identification via mass spectrometry

IP proteins in solution were analyzed at the Taplin Biological Mass Spectrometry Facility (Harvard Medical School, Boston, MA, USA). Prior to analysis, gel bands were excised, dehydrated in acetonitrile, and subsequently dried using speed‐vac and rehydrated in a solution of 50 mM ammonium bicarbonate and 12.5 ng/µL sequencing‐grade trypsin (Promega) at 4°C. Proteins were extracted upon washing in 50 mM ammonium carbonate solution at 37°C and digested with trypsin overnight at 37°C. The following day, the ammonium bicarbonate solution was removed, and samples were washed in a solution of 50% acetonitrile and 1% formic acid.^13^ Proteins were separated using Nano‐scale reverse‐phase high‐performance liquid chromatography (HPLC) LTQ Orbitrap Velos Pro (Thermo Fisher Scientific) with a capillary column and a mobile phase consisting of acetonitrile and formic acid. Detection of the mass spectra was obtained using an electrospray ionization method. Ionized protein fragments were analyzed using a database of known peptide sequences using Sequest^14^ (Thermo Fisher Scientific).

### 2.7 Bioinformatic analysis of mass spectrometry data

The csv (comma‐separated values) files with sum intensities for ApoE3 WT and Ch interactors were read into R 4.2.3 (R core team, https://www.R‐project.org/). The log2 values for Ch/WT were determined. The F‐test was employed to test sample variance. T‐tests were performed separately depending on whether the variance was statistically equal or unequal. Corrected *p*‐values were estimated using the qvalue() function. Tables were written to tab‐delimited text and imported into Excel. The significant protein list comparison was done using the ListDiff platform (http://www.listdiff.com). We used molBioTools (https://molbiotools.com) to plot Venn diagrams. The obtained protein list from mass spectrometry was used for gene ontology (GO) pathway enrichment analysis, combining two R packages, ClusterProfiling and pathview, using the SRPlot web server (https://www.bioinformatics.com.cn/srplot). Additionally, the list of interactor proteins was analyzed using QIAGEN ingenuity pathway analysis (IPA).^15^ The Diseases and Biological Functions Analysis within IPA identified the biological functions and/or diseases that were most significant from the dataset. Molecules from the dataset that bound both ApoE3 WT and ApoE3Ch and were associated with biological functions and/or diseases in the QIAGEN Knowledge Base were considered for further analysis. Cutoffs were not applied.

### 2.8 ApoE3‐tau immunoprecipitation assay

Recombinant human tau‐441 (2N4R, rPeptide, cat. T‐1001‐2) was incubated in binding buffer (NEBExpress, cat. S1423S) for 5 h at a nominal concentration of 1.2 µM (which was calculated based on the molecular weight of monomeric tau: 65 kDa) with His‐tagged ApoE3 or ApoE3Ch recombinant proteins and eluted with nickel beads following the same protocol previously described for the mouse brain immunoprecipitation. To ensure reproducibility, we repeated the experiment four times. Western blotting of the IP fraction using tau‐5 and anti‐His tag antibodies was used to detect the amount of tau protein bound to ApoE3 variants under reducing conditions.

### 2.9 Tau oligomerization assay

His‐tagged ApoE3 or ApoE3Ch proteins (*E. coli* or HEK‐derived) were incubated with recombinant monomeric tau‐441 (2N4R isoform; rPeptide, cat. T‐1001) or preformed fibrils (tau^PFF^, rPeptide, cat. TF‐1001‐2) at 1.47 µM for either 5 h or 5 days. Both tau proteins were produced in *E. coli* by rPeptide. Preformed protofibrils (PFFs) were validated by the manufacturer for their size using transmission electron tomography and Thioflavin T assay by rPeptide. For the oligomerization assay, we used either tau monomers or protofibrils and tested the changes in the aggregation pattern of the proteins, either in the absence or in the presence of ApoE variants, via Western blotting under nonreducing conditions. Western blotting using tau‐5 (Abcam, cat. ab80579) and anti‐His‐tag (Abcam, cat. ab14923) antibodies was used to ascertain the oligomerization of tau by assessing molecular weight, are reported in the Western blotting section.

### 2.10 In vitro tau aggregation and phosphorylation in tau biosensor cells

Tau RD P301S FRET Biosensor cells (ATCC, Manassas, VA, cat. CRL‐3275) were cultured in DMEM with glutaMAX (Gibco, Waltham, MA, cat. 10566016) and 10% fetal bovine serum (FBS) until confluency. To measure FRET event levels, 40.000 cells per well were seeded in a 48‐well plate and incubated overnight. tau aggregation was induced with 0.1 µM of Human tau‐441 PFFs (2N4R isoform; ACRO Biosystems, cat. TAU‐H5115) that had been validated by ACRO Biosystems using Thioflavin T assay, electron microscopy, and cell‐based assay for bioactivity. Cells were treated simultaneously with either ApoE3 WT, ApoE3Ch, or nontreated. Cells without any treatment and induction of tau aggregation were used as controls. Proteins were administered with Lipofectamine 2000 in OptiMEM (Gibco, cat. 31985070) as follows: 3 µL of Lipofectamine 2000 were mixed with 57 µL of OptiMEM and incubated for 5 min. Then, the liposomal complex was added to 60 µL of ApoE3 variants and incubated for 30 min at room temperature before adding it to each well. 1 h later, 160 µL of fresh OptiMEM was added for a total of 400 µL. The number of FRET events (nFRET) was measured using a BioTek SYNERGY H1 microplate reader (Agilent, Santa Clara, CA) by recording the fluorescence at an excitation wavelength of 430 nm and emission wavelength of 541 nm. The signal was normalized to the signal of the cyan fluorescent protein (CFP) and yellow fluorescent protein (YFP) channels according to the following equation:

$$nFRET=\frac{F_{\mathrm{FRET}}}{\sqrt{F_{\mathrm{CFP}}+F_{\mathrm{YFP}}}}$$

where $F_{\mathrm{FRET}}$ is the fluorescence of the FRET channel, $F_{\mathrm{CFP}}$ and $F_{\mathrm{YFP}}$ are the fluorescence signal of the CFP and YFP channels, respectively.

Live cell imaging was acquired in a Leica SP8 inverted microscope (Leica, Germany). 3 × 3 tile scan and z‐stack were performed. Images were deconvoluted in Huygens Essential (SVI, Netherlands).

### 2.11 Intravitreal injections of recombinant ApoE variants in mice

Intravitreal injections were performed on both WT C57BL/6J and B6;C3‐Tg(Prnp‐MAPT*P301S)PS19Vle/J mice as previously reported.^11^ All procedures adhered to the Schepens Eye Research Institute Institutional Animal Care and Use Committee (IACUC)‐approved protocol. 2 µL injections of either vehicle (PBS, pH 7.4) or various ApoE isoforms (ApoE3, ApoE3Ch, ApoE4, or ApoE4Ch) at a concentration of 50 µg/mL were delivered to the mice. 48 h later, mice were euthanized via saturating CO_2_ and cervical dislocation and eyes were enucleated and fixed overnight in 4% paraformaldehyde at 4°C. The fixed eyes were then washed twice with PBS for 5 min before retinal dissection and staining. Subsequently, retinas were blocked using a solution containing 3% donkey serum, 0.1% Triton X‐100, and 1% bovine serum albumin (BSA) in PBlec buffer. The blocking step was performed at 4°C with gentle shaking for 48 h. Primary antibodies were then applied overnight at 4°C with gentle shaking. The following primary antibodies were used: Isolectin GS‐IB4 (1:200, Thermo Fisher Scientific, cat. I21411); anti‐phospho‐tau clone AT8 (1:500, Thermo Fisher Scientific, cat. MN1020); anti‐phospho‐tau Ser396 (1:1000, Thermo Fisher Scientific, cat. 44‐752G) and anti‐His‐Tag (1:1000, Thermo Fisher Scientific, cat.  PA1‐983B). Slides were then washed twice with PBS incubated with DAPI ((4′,6‐diamidino‐2‐phenylindole; 1:1000 in PBS, Millipore Sigma, cat. 10236276001) for 15 min and then washed every 15 min with PBS for 45 min in total prior to mounting the samples Vectashield fluoromount (Vector Laboratories, Newark, CA, cat. H‐1000‐10).

### 2.12 Enzyme‐linked immunosorbent assay

We used an enzyme‐linked immunosorbent assay (ELISA) to determine binding between ApoE variants and preformed tau protofibrils by testing serially diluted concentrations of preformed tau fibrils between 0.2 nM and 0.77 µM incubated with 0.0025 µg/µL of ApoE‐coated plates in TBS enriched with 2 mM CaCl_2_. After 18 h incubation at 4°C under gentle shaking, the plate was blocked with 3% BSA in TBT‐T enriched with 2 mM CaCl_2_ for 1 h at room temperature. Subsequently, changes in tau binding were determined by incubating with anti‐tau antibody (clone TAU‐5; Abcam, cat. ab80579) for 2 h, followed by 1 h incubation with goat anti mouse‐horseradish peroxidase (HRP) conjugated secondary antibody (Abcam, cat. ab205719). Colorimetric reaction was induced upon incubating the plate for 20 min in the dark with 3,3′5,5′‐tetramethylbenzidine substrate (TMD; R&D Systems, cat. DY999B). Between steps, plates were washed three times with TBS‐T enriched with 2 mM CaCl_2._ Optical densities were detected spectrophotometrically at 450 nm using a Biosystem H1 bioanalyzer.

### 2.13 Statistical analyses

We performed the Kolmogorov–Smirnov normality test for normal distribution, F‐Test, and T‐Test on the mass spectrometry data. A right‐tailed Fisher's exact test was used to calculate a *p*‐value determining the probability that each biological function and/or disease assigned to that data set is due to chance alone. A *z*‐score was calculated to indicate the likelihood of an increase or decrease in that function. Two‐way analysis of variance (ANOVA) was performed to analyze the number of bound proteins (variables: ApoE3 variants, source of the recombinant proteins), followed by Fisher's least significant difference (LSD) test, while one‐way ANOVA was performed to compare the IP fractions of ApoE3 variants in the presence of Dkk1 recombinant protein, the FRET levels, and tau aggregates. Both ANOVA analyses were conducted using GraphPad Prism 10.

## 3 RESULTS

### 3.1 Proteomic characterization of ApoE3 WT and ApoE3Ch interacting partners

**FIGURE 1:**
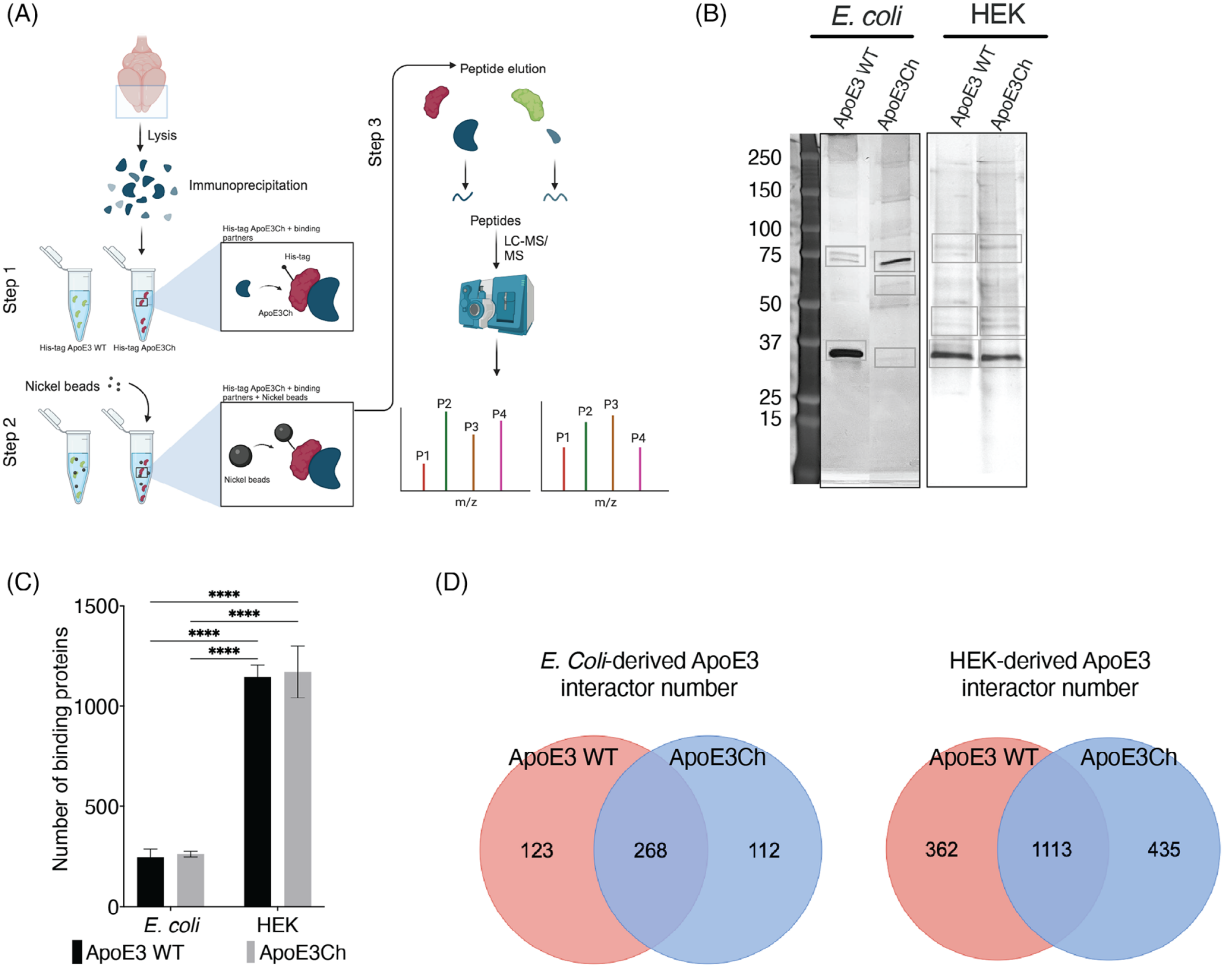
Proteomic analyses of ApoE3 variants. (A) Experimental design of the ApoE co‐immunoprecipitation experiments. (B) Representative silver stain of the IP fractions using recombinant ApoE3 WT and ApoE3Ch proteins from *E. coli* (left blot) or HEK‐derived (right blot). (C). Number of interactor proteins identified by mass spectrometry (*n* = 3, independent pull‐down experiments). (D) Pie charts summarizing the number of unique and shared proteins binding to ApoE3 WT and ApoE3Ch. ApoE3, apolipoprotein E3; ApoE3Ch, apolipoprotein E3 Christchurch variant; HEK, human embryonic kidney; IP, immunoprecipitated; WT, wild‐type.

We used mouse frontal cortex homogenates of 3‐month‐old female mice (*n* = 3) to determine the effect of the ApoE3 Christchurch variant on the protein–protein interactions between recombinant human His‐tagged ApoE3 and brain‐derived proteins (Figure 1). The frontal cortex was chosen due to its relative abundance of astrocyte and microglial cell populations, which is crucial given glia's involvement in ApoE production,^16^ and that several biochemical pathways are shared between mouse and human brain.^17^, ^18^

Considering that the interaction with the His‐tagged proteins might be affected by post‐translational modifications (PTMs) occurring in mammalian cells, we used “unmodified” ApoE3 produced recombinantly in *E. coli* and ApoE3 purified from mammalian cells (HEK cells) to detect relevant interactions mediated by glycosylation or lipidation levels. The proteins eluted from the IP samples were qualitatively detected using silver staining (Figure 1B).

The analysis of the pulled‐down fractions by liquid chromatography coupled to mass spectrometry (LC‐MS) revealed that, despite the presence of the Ch variant, there was no significant difference between the total number of bound interactors. We calculated that an average of 1146 interactors were bound to HEK‐derived ApoE3 WT, while an average of 1170 interactors bound to ApoE3Ch (Figure 1C). However, we observed that the number of interactors bound to *E. coli*‐derived ApoE3 variants was significantly lower than the numbers associated with HEK‐derived variants, suggesting that the PTMs of ApoE3 are important in promoting the ability of ApoE to interact with other proteins. We further analyzed the number of proteins that were bound to either *E. coli*‐derived or to HEK293‐derived variants. Within these clusters of interacting proteins, we quantified the total number of interactors that were either bound uniquely to ApoE3 WT or ApoE3Ch or the ones that were shared between the ApoE3 variants. Analysis of mammalian‐derived PTMs revealed an increase in the number of unique interacting partners from 123 to 362 for ApoE3 WT and from 112 to 435 for ApoE3Ch and the number of shared interactors from 268 to 1113 (Figure 1D). Overall, our data suggest that PTMs impact the number of interacting partners more profoundly than the presence of the Ch variant does.

### 3.2 Gene ontology (GO) and pathway analyses reveal novel HSPG‐independent pathways downstream of ApoE

**FIGURE 2:**
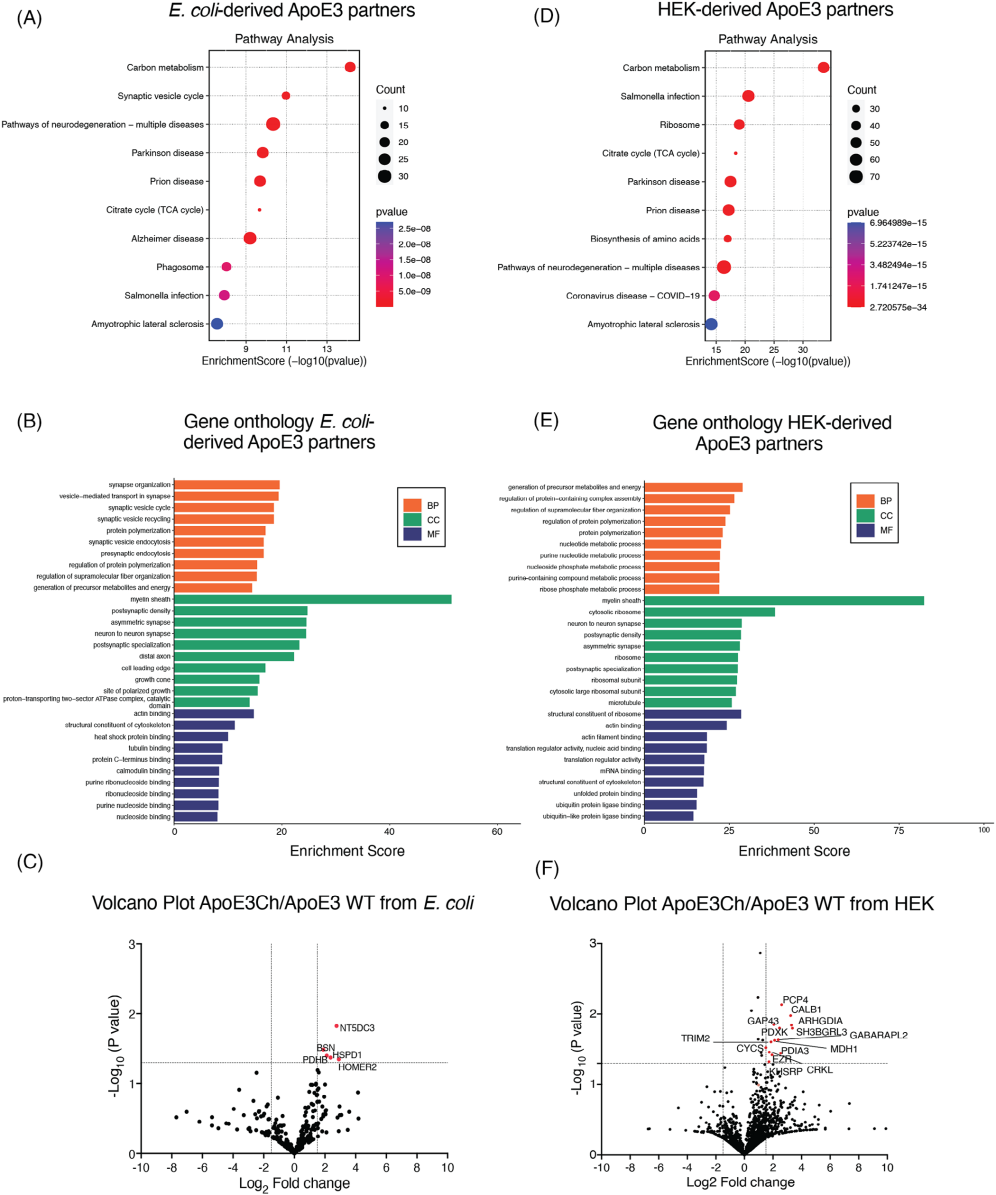
GO analyzes of interacting partners of ApoE3 WT and ApoE3Ch from different sources. (A) Pathway enrichment analysis based on the fold change of the proteins binding both ApoE3 WT and ApoE3Ch, E. coli‐derived (upper graph) and HEK‐derived (bottom graph). (B) BP, CC, and MF ontologies of ApoE3 E. coli (upper graph) and HEK‐derived ApoE3 binding partners (bottom). (C) Volcano plots showing log2 ratios of interactor protein binding (ApoE3Ch/ApoE3 WT). Significant interactors are shown in red. ApoE3, apolipoprotein E3; ApoE3Ch, apolipoprotein E3 Christchurch variant; BP, biological process; CC, cellular component; GO, gene ontology; HEK, human embryonic kidney; MF, molecular function; WT, wild‐type.

To investigate the physiological relevance of the ApoE3 interacting partners, we conducted a GO analysis for the interactors associated uniquely with ApoE3 WT or ApoE3Ch (Figure 2A–E) and the interactors binding both ApoE3 variants. The *sum* intensity of the fold change of the ApoE3 WT and ApoE3Ch interactors was used for pathway enrichment analysis. We found that carbon metabolism was the most significant pathway involving the ApoE3 interactors from *E. coli*, followed by synaptic vesicle cycle, pathways of multiple neurodegenerative diseases, Parkinson's disease, prion disease, citrate cycle (TCA cycle), Alzheimer's disease, phagosome, Salmonella infection and amyotrophic lateral sclerosis (Figure 2A). The three‐ontology (biological process, cellular component, and molecular function) analyses of the *E. coli*‐derived group showed that the most relevant biological processes are related to the synapses, including the organization, transport, recycling, endocytosis, and regulation of protein polymerization. The major cellular components are the myelin sheath, different synaptic compartments, and the growth cone. The major molecular function is binding and structural constituent of the cytoskeleton (Figure 2B). We used Volcano plots to analyze the mass spectrometry data to highlight the significantly changing interactors between ApoE3Ch and ApoE3 WT (Figure 2C). We found five statistically significant levels of protein fragments that were detected in the IP fractions encoded by NT5DC3, BSN, PDHB, HSPD1, and HOMER2 genes which have roles in 5′‐nucleotidase activity for dephosphorylation,^19^ transcriptional regulation, organization and maintenance of the presynaptic release apparatus,^20^ tau propagation and neurotoxicity,^21^ ATP production,^22^ chaperones^23^ and receptor trafficking at the synapses^24^ (Table S1). Interestingly, all the significant proteins found in the volcano plot analysis were more abundant in ApoE3Ch.

Pathway analysis of the HEK‐derived ApoE3 interactors showed similar pathways to the *E. coli*‐derived, except for synaptic vesicle cycle, AD, and phagosome (Figure 2A). The most relevant pathways were carbon metabolism, followed by Salmonella infection, Ribosome, Citrate cycle (TCA cycle), Parkinson's disease, prion disease, biosynthesis of amino acids, pathways of neurodegeneration‐multiple diseases, coronavirus disease 2019 (COVID‐19), and amyotrophic lateral sclerosis (Figure 2D). Compared to the *E. coli*‐derived results, ribosome, biosynthesis of amino acids and coronavirus disease were present only in the HEK‐derived ApoE3 interactors. Furthermore, the three‐ontology analysis showed that the HEK‐derived ApoE3 interactors participate in different biological processes, such as the generation of precursor metabolites and energy, and the regulation of several processes, including protein‐containing complex assembly, supramolecular fiber organization and protein polymerization. The cellular components of these proteins are the synapse, ribosome, and microtubules. The main molecular function is contributing to the structure of the ribosome and actin binding, nucleic acid binding, mRNA binding, unfolded protein binding, and ubiquitin protein ligases binding (Figure 2E). Volcano plot analysis of the HEK‐derived ApoE3 interactors showed a significant increase in the number of proteins compared to the *E. coli*‐derived group. Specifically, calmodulin regulator protein (Pcp4) and calbindin (Calb1) were the proteins with more fragments binding ApoE3Ch than ApoE3 WT. The other significantly increased proteins in ApoE3Ch are: Arhgdia, Gap43, Pdxk, Sh3grl3, Gabarapl2, Mdh1, Trim2, Cycs, Crkl, Pdia3, Ezr, Khsrp (Figure 2F and Table S2). Many of these proteins are known to have a role in axonal transport and neurogenesis or have a known pathophysiological role in neurodegenerative diseases.^25^, ^26^, ^27^ All the significant proteins found in the volcano plot analysis were more abundant in ApoE3Ch (positive fold change).

**FIGURE 3:**
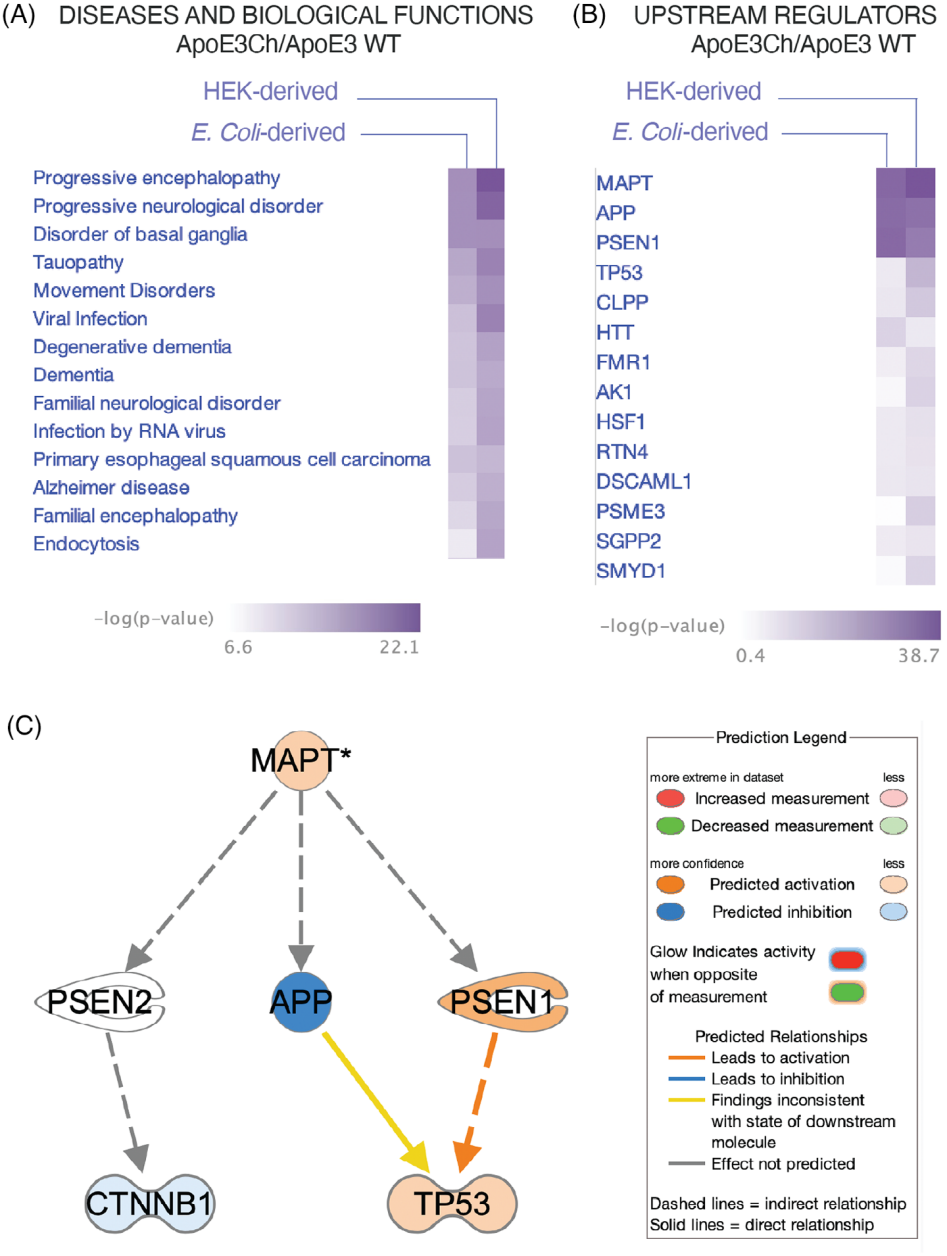
Ingenuity pathway analysis. (A) Comparative analysis of disease and biological function in ApoE3Ch versus ApoE3 WT interactors highlighting brain‐related disorders. (B) Comparison analysis of upstream regulators in ApoE3Ch versus ApoE3 WT interactors. (C) Pathway diagram with MAPT as the top upstream regulator showing indirect link to PSEN1, PSEN2, and APP, which are in turn linked to CTNNB1 and TP53. APP, amyloid precursor protein; ApoE3, apolipoprotein E3; ApoE3Ch, apolipoprotein E3 Christchurch variant; MAPT, microtubule‐associated protein tau; PSEN, presenilin; WT, wild‐type.

Comparisons of the IPA of diseases and biological functions showed diseases related to neurological disorders, including tauopathy (Figure 3A).

IPA examination of the predicted upstream regulators confirmed that microtubule‐associated protein tau (MAPT), APP, and PSEN1 are the standard upstream regulators for all ApoE variants (Figure 3B). These results indicate that tau regulates the expression, transcription, or phosphorylation of 80 proteins from the mass spectrometry dataset. tau was detected as MAPT fragments in the mass spectrometry data in all ApoE3 variants from either *E. coli* or HEK (Tables S3 and S4, Figure S2A–E). Mechanistic network analysis was employed to discover plausible sets of connected upstream regulators that can work together to elicit the gene expression changes observed in the dataset (Figure 3C).

### 3.3 Christchurch variant in ApoE3 enhances anti‐aggregation of tau in vitro

**FIGURE 4:**
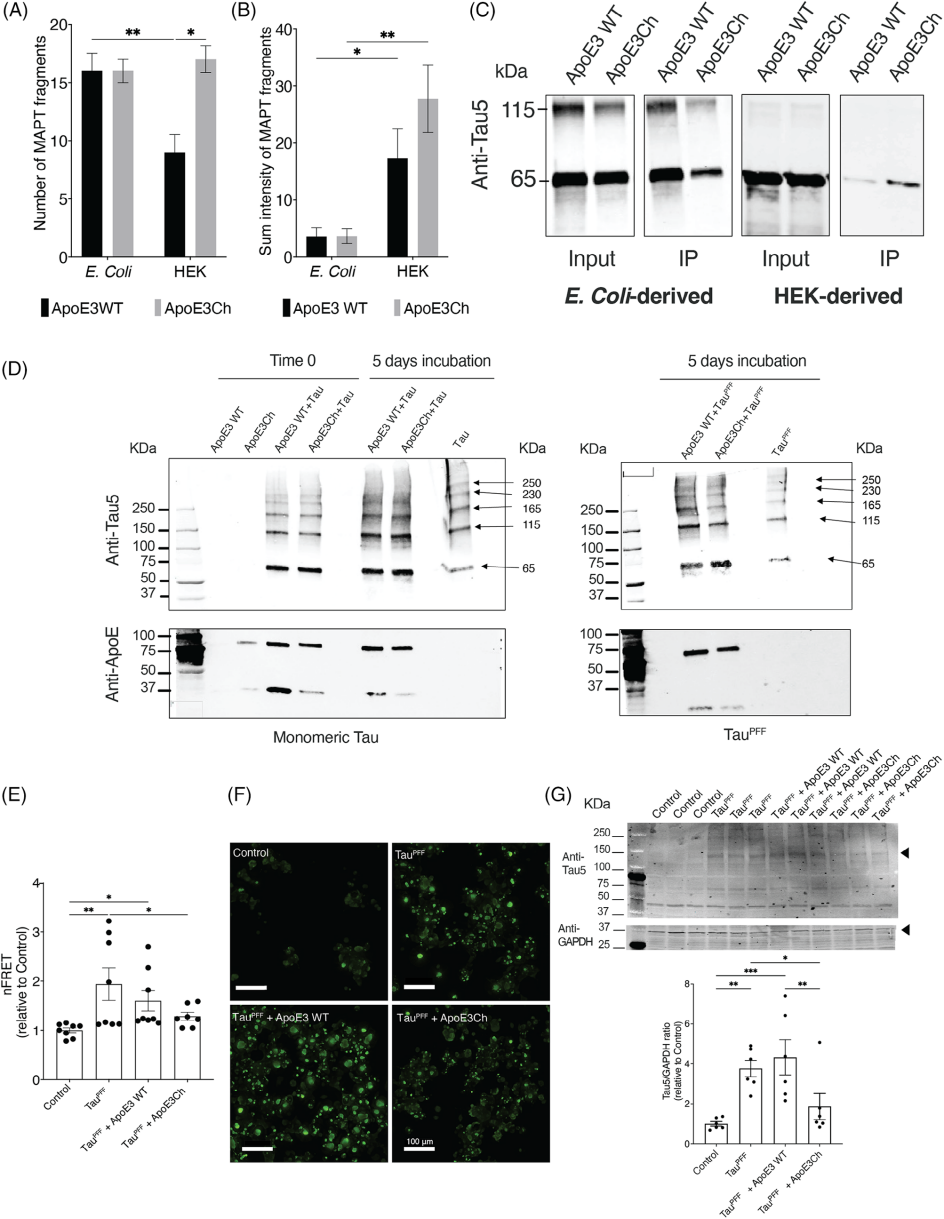
ApoE3 WT and ApoE3Ch differentially interacts with tau. (A, B) Number of MAPT fragments and sum intensity obtained via mass spectrometry analysis (*p ≤ 0.05, **p ≤ 0.01, Two‐way ANOVA, Fisher's LSD post‐hoc test, *n* = 3). (C) Co‐immunoprecipitation of human tau recombinant protein using His‐tag ApoE3 WT and ApoE3Ch proteins and detected via WB using anti‐tau antibody. (D) Representative Western blot of ApoE3 WT and ApoE3Ch after incubation with monomeric recombinant human tau or tau PFFs, arrows point the different tau aggregate bands. (E) Levels of FRET signal in tau RD P301S FRET Biosensor cells stimulated with tau PFF and treated with APOE3 variants (*p ≤ 0.05, **p ≤ 0.01, one‐way ANOVA, Fisher's LSD post‐hoc test, *n* = 6). (F) Scale bar: 100 µm. Representative maximum intensity projections of the live imaging acquisition of the tau aggregates in the tau RD P301S FRET Biosensor cells stimulated with tau PFFs and treated with the ApoE3 variants. (G) WB of tau aggregates in tau RD P301S FRET Biosensor cells treated with ApoE3 variants and ratio quantification between the total tau aggregates at ∼ 150 kDa of molecular weight and GAPDH, quantified bands are indicated by an arrowhead. (*p ≤ 0.05, **p ≤ 0.01, ***p ≤ 0.001, one‐way ANOVA, Fisher's LSD post‐hoc test, *n* = 6). ANOVA, analysis of variance; ApoE3, apolipoprotein E3; ApoE3Ch, apolipoprotein E3 Christchurch variant; GAPDH, glyceraldehyde‐3‐phosphate dehydrogenase; LSD, least significant difference; PFF, preformed protofibrils; WB, Western blotting; WT, wild‐type.

We focused our attention on MAPT (tau), given that it is a hallmark of AD^28^ and both a predicted upstream regulator and a detected hit in our mass spectrometry data. Although MAPT was not a statistically significant protein in the Volcano plot analysis, we confirmed that HEK‐derived ApoE3Ch was associated with a higher number of tau‐fragments when we used ApoE3 variants to pull down brain fraction protein (Figure 4A–C). Moreover, a comprehensive list of interactors supporting MAPT prediction as the top upstream regulator is reported in Table S5.

We further investigated whether the direct interaction between ApoE and tau was impacting the aggregation propensity of tau (Figure 4D). When using recombinant human tau protein, we observed increased levels of monomeric tau associated with HEK‐derived ApoE3Ch, which was consistent with our mass spectrometry data. When we tested the effect of ApoE3 variants on tau aggregation by comparing the effect of incubating either monomeric tau or tau PFF up to 5 days with ApoE variants, we observed that ApoE3Ch had a direct antiaggregating effect on tau as compared to ApoE3 WT (Figure 4D). Further, we confirmed an increased binding between ApoE3Ch and preformed tau fibrils via ELISA (Figure S3A–B) that was not detectable via bio‐layer interferometry (Figure S3C–D). These findings support the hypothesis of a transient interaction between ApoE3Ch  and tau, a finding consistent with previously reported evidence of a direct interaction between ApoE and tau.^29^, ^30^, ^31^ Similarly, we have previously demonstrated a similar ApoE3Ch variant effect on amyloid beta.^5^ Our findings demonstrate a previously unknown direct antiaggregating effect of ApoE3Ch on tau and interaction with preformed tau aggregates.

We further validated the effect of the interaction between ApoE3 variants in an in vitro cell model, the Tau RD P301S FRET Biosensor cells.^32^ This stable cell line produces tau P301S conjugated with either CFP or YFP tag simultaneously, and a FRET signal is generated when the tau protein is aggregated. We induced tau aggregation by incubating with commercially available tau PFF and evaluated the antiaggregating capability of the ApoE3 variants. FRET levels were significantly lower in cells treated with ApoE3Ch variant compared with the cells treated only with tau fibrils (Figure 4E). We observed the auto‐florescence aggregates via live‐cell imaging: cells treated with tau PFF alone and tau PFF co‐incubated with ApoE3 WT showed an increase in tau aggregates compared with control, and a decrease was observed in the cells treated with ApoE3Ch. (Figure 4F). Western blot of the tau RD P301S FRET Biosensor cell lysed confirmed the reduction in tau aggregation. We observed an enrichment of the 150 kDa band of total tau aggregates when the cells were treated with tau PFF as compared to untreated. The presence of ApoE3Ch significantly reduced the aggregation of tau, as confirmed by the reduced levels of the 150 kDa band (Figure 4G). Moreover, we observed a tendency toward reduction of the high molecular weight oligomers and monomers of phospho‐tau when normalized against glyceraldehyde‐3‐phosphate dehydrogenase (GAPDH), used as a loading control (Figure S4A–D).

### 3.4 ApoE3Ch modulates tau phosphorylation in a mouse model of tau pathology

**FIGURE 5:**
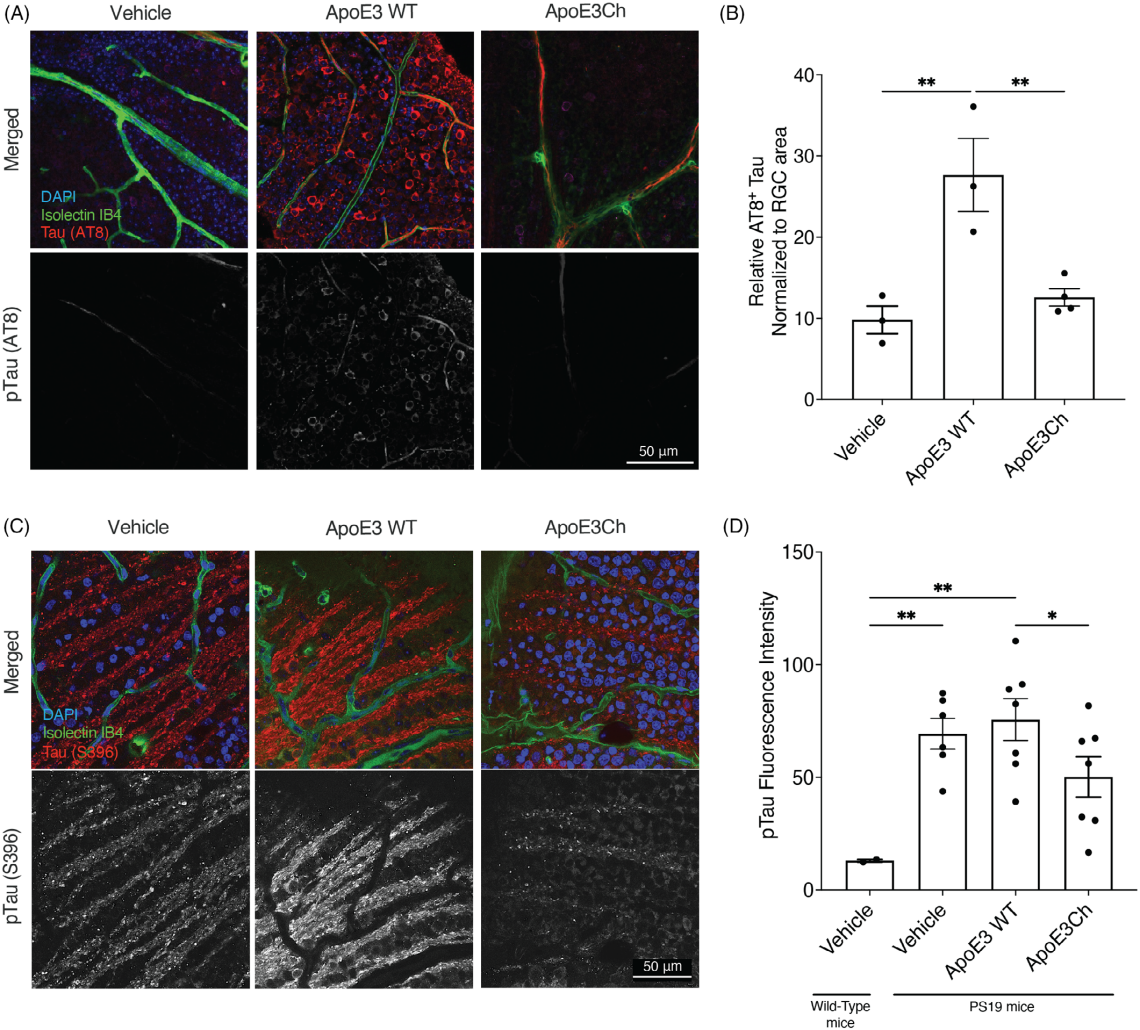
Effect of Christchurch variant on ApoE‐induced tau pathology in the mouse retina. (A) Representative immunofluorescence of subretinally injected MAPT P301S tau transgenic mice with recombinant ApoE3 and ApoE3Ch variants, as compared to vehicle, showing reduced levels of AT8‐positive cells in the presence of the Ch variant. (B) Relative AT8+ tau levels in dissected retina isolated from mice treated with either vehicle, ApoE3, or ApoE3Ch, showing that in the presence of ApoE3Ch, AT8+ cells are significantly reduced (**p < 0.005, One‐way ANOVA, *n* = 3‐4). (C) immunofluorescence staining of intravitreal injected 9 months old MAPT P301S tau mice with recombinant ApoE3 and ApoE3 Ch proteins, showing the reduced levels of ptau S396‐positive cells in the presence of the Ch variant. (D) Intravitreal injection of ApoE3 Ch in the retina significantly decreased phosphorylation of tau (* = *p* < 0.05, one‐way ANOVA, *n* = 2–5). Vehicle treated wild‐type mice were used as control for MAPT P301S mice (PS19). For both panels A and C, nuclei are stained with DAPI in blue, vascular formation is stained with Isolectin (IB4) in green and tau phosphorylation are stained with AT8 anti tau (A) or ptau S396 antibody (C) in red. Scale bar = 50 µm. ANOVA, analysis of variance; ApoE3, apolipoprotein E3; ApoE3Ch, apolipoprotein E3 Christchurch variant; DAPI, 4′,6‐diamidino‐2‐phenylindole.

We previously reported that ApoE3 triggers early onset tau pathology in the retina of MAPTP301S tau transgenic mouse models, and that ApoE‐induced tau pathology can be successfully rescued by anti‐ApoE antibodies that target the HSPG‐domain of ApoE3.^11^ Using our previously established retina model, we tested the effect of intravitreally injected ApoE3 WT and ApoE3Ch variants in 40‐day‐old MAPT P301S mice. We observed that ApoE3 WT significantly induced the phosphorylation of tau, measured with an anti‐phosphorylated‐tau (clone AT8) antibody (Figure 5A, B) in 40‐day‐old MAPT P301S mice. ApoE3Ch maintained low levels of tau phosphorylation similar to that of the vehicle. Moreover, we observed that recombinant ApoE3 proteins colocalize with the ptau signal (AT8) in the mouse retina (Figure S5C), indicating that the intravitreal injection effectively delivers the proteins into the retinal layers.

We also evaluated the levels of phosphorylation in mice injected with ApoE4 variants (Figure S5A). ApoE4 WT caused severe damage in the mouse retinas, making quantification unfeasible. However, the incorporation of the Christchurch variant in the ApoE4 protein showed a reduction of tau pathology compared with the wild‐type protein.

In a second experiment, we used 9‐month‐old MAPT P301S mice with manifesting pathology (Figure 5C, D). We quantified changes in tau phosphorylation as a measure of ApoE‐induced pathology using an anti‐phospho‐tau Ser396 specific antibody to measure the effect of ApoE3Ch on ApoE‐induced pathology. Phosphorylation of tau Ser396 correlates with early tau pathology in this mouse model.^33^ Immunofluorescence analyses of intravitreally injected retina showed a significantly reduced levels of phospho‐tau in the presence of ApoE3Ch as compared to ApoE3 WT injected mice and reduced pathology spread homogenously across the retina (Figure S5B).

### 3.5 ApoE and Dkk1 interaction as a novel mechanism of protection

In addition to employing an unbiased approach, we performed a candidate protein analysis based on upstream regulators of the HEK‐derived ApoE3Ch interactors (Table S6) and previous studies investigating potential protective mechanisms of Christchurch.^34^ Our earlier work has shown that the ApoE3Ch variant amplifies Wnt signaling through a neomorphic effect, prompting us to hypothesize that protein–protein interactions with components of this pathway may play a central role.^35^ Dickkopf1 (Dkk1) is the most well‐characterized inhibitor of Wnt signaling, and its production increases in aging and dementia models.^36^ We therefore hypothesized that the neomorphic amplification of Wnt signaling by ApoE3Ch is mediated through an interaction between ApoE3Ch and Dkk1, which could sequester the Dkk1 inhibitory effect.

To test this hypothesis, we used co‐immunoprecipitation assays to confirm the interaction between recombinant ApoE variants and Dkk1. Our data show that both ApoE3 WT and ApoE3Ch interact with Dkk1; however, the interaction is more robust for ApoE3Ch (Figure S6A–C), supporting our earlier findings in iPSC models. We further validated this interaction using isothermal titration calorimetry (ITC), which revealed that the ApoE3 WT‐Dkk1 interaction is initially endothermic, transitioning to an exothermic interaction over time with higher concentrations of ApoE3 WT (Figure S6D–G). Due to the pro‐aggregating nature of ApoE3Ch, we were unable to titrate the interaction between Dkk1 and ApoE3Ch via ITC.

In parallel, we examined the ApoE3Ch‐dependent amplification of Wnt3a signaling in the presence of Dkk1 to assess its biological relevance. Dkk1 significantly reduced Wnt signaling by more than 50%, but in the presence of ApoE3Ch, Wnt signal remained unchanged (Figure S6H). These results suggest that ApoE3Ch may block the inhibitory effect of Dkk1, thus maintaining Wnt/β‐catenin pathway signaling.

## 4 DISCUSSION

ApoE and its variants play a significant role in AD, which can be either detrimental or protective against this disease. ApoE is known to have a critical role in lipid transport, synaptic function, and regulation of either the clearance or spreading of neurotoxic Aβ and tau aggregates via the HSPGs‐Apolipoprotein receptor pathway.^37^, ^38^, ^39^, ^40^ Our group and others showed that changes in the interaction with HSPGs correlate with the gain of toxicity of ApoE4 and possibly with the loss of function of protective variants ApoE2, ApoE3Ch, and ApoE‐Jacksonville.^5^, ^41^ ApoE3 variants are known to bind to HSPGs in either lipidated or non‐lipidated states and can interact with the family of apolipoprotein receptors.^42^ We previously reported the protective effect of the *APOE3C*h variant and found reduced levels of tau accumulation in the ADAD‐protected case. Based on the known ApoE biology, we hypothesized that a loss of function of ApoE3Ch due to reduced HSPG binding was one possible mechanism of protection against tau pathology.^5^ In the current study, we show that posttranslational modifications such as glycosylation and lipidation might also facilitate direct interaction between ApoE3 and other neuronal proteins, and that this interaction can occur via alternative interactions. PTMs of ApoE3 play a crucial role in modulating its interactions,^43^ and while this study focuses on primary interactors, additional data on PTMs—including phosphorylation and glycosylation—should be considered in future investigations.

We found more proteins that are interactors for ApoE3Ch, thus leading to the hypothesis that a gain of functional protein–protein interactions leads to the upregulation of neuroprotective functions in the presence of a loss of HSPG‐interaction. Interestingly, these regulators are all well‐known relevant genetic risk factors of AD and related dementias.^44^

One of the most remarkable findings in the *APOE3Ch* case was the reduced tau pathology.^5^ tau normally mediates the stability and assembly of microtubules through dynamic changes in phosphorylation.^45^ However, hyperphosphorylated tau is upstream of pathological changes leading to misfolding and self‐assembly of tau into neurofibrillary tangles (NFTs).^46^ Therefore, in this study, we tested whether tau interacts differently with ApoE3 WT and ApoE3Ch variants. Interestingly, we found that ApoE3Ch binds with a stronger affinity to tau and that this interaction leads to reduced tau aggregation, thus supporting the hypothesis of alternative pathways leading to the direct cross‐seeding between ApoE3Ch and tau that results in smaller “off‐pathway” tau aggregates that might be less toxic. Our findings have been recently validated in parallel studies by Chen et al.^47^

It is well documented that Wnt signaling is hamstrung in the aged and neurodegenerating brain.^48^ We have previously reported on the role of ApoE3Ch in enhancing Wnt3a signaling in a reporter cell line, and that this effect was not due to a direct interaction with the receptor complex.^35^ Our study here identified a novel Dkk1‐ApoE3Ch protein–protein interaction, which may provide a hint at the full mechanism of Wnt signaling upregulation, possibly via a gain of interaction in the presence of the Christchurch variant. Dkk1 is an antagonist of the Wnt/β‐catenin pathway, thus, an increased effect of Dkk1 has been proposed as one of the contributors to AD pathogenesis by promoting Aβ‐induced synaptic loss, tau hyperphosphorylation and neuroinflammation both in vitro, and ex vivo.^49^, ^50^, ^51^ Our data suggest that ApoE interacts with Dkk1 with low affinity. However, this interaction may be physiologically relevant because of the high abundance of ApoE in biological fluids where ApoE may titrate the levels of Dkk1. Importantly, specific ApoE isoforms may differentially modulate Dkk1 function by virtue of their different concentrations in biological fluids. For instance, it has been reported that ApoECh accumulates more in biological fluids compared to other ApoE isoforms.^52^, ^53^, ^54^ Consistently, extensive literature reported on differential levels of ApoE2 versus ApoE4 in both cerebral spinal fluid (CSF) and blood.^55^, ^56^ Indeed, others have shown that tau aggregation can be modulated by several factors that can readdress the typical pathway of aggregation leading to NFTs, a hallmark of AD. Conversely, these off‐pathway aggregates form oligomeric tau strains that are not necessarily neurotoxic.^57^ Overall, our study shows a novel alternative pathway of ApoE3Ch protective effects that is not associated with HSPG interaction and that leads to a neuroprotective effect via the direct interaction with other proteins, such as tau. It is reasonable to hypothesize that the protective effect of ApoE3Ch is, at least partly, thanks to a gain of interaction with other neuronal proteins that are favored by the loss of interaction with HSPG. Our findings may bolster therapeutic strategies that enhance our previously described therapeutic antibody approach, mimicking the effects of ApoE Christchurch. We propose that the profound protective effects conferred by ApoE Christchurch result from a combination of mechanisms. Therefore, optimal protection might be achieved through therapeutic strategies that include both gain‐of‐function and loss‐of‐function approaches in a complementary manner.

## AUTHOR CONTRIBUTIONS

*Methodology*: Paula Perez‐Corredor, Said Arevalo‐Alquichire, Randall C. Mazzarino, Michael O'Hare, Andres F. Muriel‐Torres, Guido N. Vacano, William P. Miller, Joseph F. Arboleda‐Velasquez, and Claudia Marino. *Investigation*: Paula Perez‐Corredor, Said Arevalo‐Alquichire, Randall C. Mazzarino, Michael O'Hare, Andres F. Muriel‐Torres, Guido N. Vacano, William P. Miller, Lina Pineda‐Lopez, Shivani Patel, Nihat Polat, Leo A. Kim, Joseph F. Arboleda‐Velasquez, and Timothy E. Vanderleest. *Analysis*: Paula Perez‐Corredor, Said Arevalo‐Alquichire, Timothy E. Vanderleest, Robert A. Obar, and Joseph F. Arboleda‐Velasquez. *Visualization*: Paula Perez‐Corredor, Said Arevalo‐Alquichire, Timothy E. Vanderleest, and Robert A. Obar. *Writing Draft*: Paula Perez‐Corredor and Said Arevalo‐Alquichire. *Writing and Editing*: Paula Perez‐Corredor, Said Arevalo‐Alquichire, Leo A. Ki, Joseph F. Arboleda‐Velasquez, and Claudia Marino. *Conceptualization*: Joseph F. Arboleda‐Velasquez and Claudia Marino. *Supervision*: Joseph F. Arboleda‐Velasquez and Claudia Marino.

## CONFLICT OF INTEREST STATEMENT

Dr. J. Arboleda‐Velasquez is listed as co‐inventor on a patent to leverage therapeutics based on the ApoE3 Christchurch findings filed by Mass General Brigham. Drs. Arboleda‐Velasquez and Kim are co‐founders of Epoch Biotech, a company focused on developing therapeutics inspired by the Christchurch variant. All other authors have no conflicts to disclose. Author disclosures are available in the Supporting Information.

## CONSENT STATEMENT

Human samples or patient data were not used in this study.

## Supporting information



Supporting Information

Supporting Information

## References

[1] Alzheimer's Association . 2019 Alzheimer's disease facts and figures. Alzheimer's Association; 2019.

[2] Bekris LM , Yu CE , Bird TD , Tsuang DW . Genetics of Alzheimer disease. J Geriatr Psychiatry Neurol. 2010;23(4):213‐227. 21045163 10.1177/0891988710383571 PMC3044597

[3] Lalli MA , Cox HC , Arcila ML , et al. Origin of the PSEN1 E280A mutation causing early‐onset Alzheimer's disease. Alzheimers Dement. 2014;10(5):S277‐S283.e10. 24239249 10.1016/j.jalz.2013.09.005 PMC4019728

[4] Acosta‐Baena N , Sepulveda‐Falla D , Lopera‐Gomez CM , et al. Pre‐dementia clinical stages in presenilin 1 E280A familial early‐onset Alzheimer's disease: a retrospective cohort study. Lancet Neurol. 2011;10(3):213‐220. 21296022 10.1016/S1474-4422(10)70323-9

[5] Arboleda‐Velasquez JF , Lopera F , O'Hare M , et al. Resistance to autosomal dominant Alzheimer's disease in an APOE3 Christchurch homozygote: a case report. Nat Med. 2019;25(11):1680‐1683. 31686034 10.1038/s41591-019-0611-3 PMC6898984

[6] Frieden C , Garai K . Structural differences between apoE3 and apoE4 may be useful in developing therapeutic agents for Alzheimer's disease. Proc Natl Acad Sci U S A. 2012;109(23):8913‐8918. 22615372 10.1073/pnas.1207022109 PMC3384159

[7] Liao F , Yoon H , Kim J . Apolipoprotein E metabolism and functions in brain and its role in Alzheimer's disease. Curr Opin Lipidol. 2017;28(1):60‐67. 27922847 10.1097/MOL.0000000000000383 PMC5213812

[8] Futamura M , Dhanasekaran P , Handa T , Phillips MC , Lund‐Katz S , Saito H . Two‐step mechanism of binding of apolipoprotein E to heparin: implications for the kinetics of apolipoprotein E‐heparan sulfate proteoglycan complex formation on cell surfaces. J Biol Chem. 2005;280(7):5414‐5422. 15583000 10.1074/jbc.M411719200

[9] Saito H , Dhanasekaran P , Nguyen D , et al. Characterization of the heparin binding sites in human apolipoprotein E. J Biol Chem. 2003;278(17):14782‐14787. 12588864 10.1074/jbc.M213207200

[10] Husain MA , Laurent B , Plourde M . APOE and Alzheimer's disease: from lipid transport to physiopathology and therapeutics. Front Neurosci. 2021;15:630502. 33679311 10.3389/fnins.2021.630502 PMC7925634

[11] Marino C , Perez‐Corredor P , O'Hare M , et al. APOE Christchurch‐mimetic therapeutic antibody reduces APOE‐mediated toxicity and tau phosphorylation. Alzheimers Dement. 2023;20(2):819‐836. 37791598 10.1002/alz.13436 PMC10916992

[12] Wesener DA , Wangkanont K , McBride R , et al. Recognition of microbial glycans by human intelectin‐1. Nat Struct Mol Biol. 2015;22(8):603‐610. 26148048 10.1038/nsmb.3053 PMC4526365

[13] Shevchenko A , Wilm M , Vorm O , Mann M . Mass spectrometric sequencing of proteins silver‐stained polyacrylamide gels. Anal Chem. 1996;68(5):850‐858. 8779443 10.1021/ac950914h

[14] Peng J , Gygi SP . Proteomics: the move to mixtures. J Mass Spectrom. 2001;36(10):1083‐1091. 11747101 10.1002/jms.229

[15] Krämer A , Green J , Pollard J Jr , Tugendreich S . Causal analysis approaches in ingenuity pathway analysis. Bioinformatics. 2014;30(4):523‐530. 24336805 10.1093/bioinformatics/btt703 PMC3928520

[16] Wang N , Cai L , Pei X , et al. Microglial apolipoprotein E particles contribute to neuronal senescence and synaptotoxicity. iScience. 2024;27(6):110006. 38868202 10.1016/j.isci.2024.110006 PMC11167441

[17] Carlyle BC , Kitchen RR , Kanyo JE , et al. A multiregional proteomic survey of the postnatal human brain. Nat Neurosci. 2017;20(12):1787‐1795. 29184206 10.1038/s41593-017-0011-2 PMC5894337

[18] Zhang B , Zhang S , Zhang S . Whole brain alignment of spatial transcriptomics between humans and mice with brainalign. Nat Commun. 2024;15(1):6302. 39080277 10.1038/s41467-024-50608-2 PMC11289418

[19] Banditelli S , Baiocchi C , Pesi R , et al. The phosphotransferase activity of cytosolic 5'‐nucleotidase; a purine analog phosphorylating enzyme. Int J Biochem Cell Biol. 1996;28(6):711‐720. 19927594 10.1016/1357-2725(95)00171-9

[20] Gundelfinger ED , Reissner C , Garner CC . Role of bassoon and piccolo in assembly and molecular organization of the active zone. Front Synaptic Neurosci. 2015;7:19. 26793095 10.3389/fnsyn.2015.00019 PMC4709825

[21] Martinez P , Patel H , You Y , et al. Bassoon contributes to tau‐seed propagation and neurotoxicity. Nat Neurosci. 2022;25(12):1597‐1607. 36344699 10.1038/s41593-022-01191-6 PMC9708566

[22] Kikuchi D , Minamishima YA , Nakayama K . Prolyl‐hydroxylase PHD3 interacts with pyruvate dehydrogenase (PDH)‐E1β and regulates the cellular PDH activity. Biochem Biophys Res Commun. 2014;451(2):288‐294. 25088999 10.1016/j.bbrc.2014.07.114

[23] Campanella C , Pace A , Caruso Bavisotto C , et al. Heat shock proteins in Alzheimer's disease: role and targeting. Int J Mol Sci. 2018;19(9):2603. 30200516 10.3390/ijms19092603 PMC6163571

[24] Parisiadou L , Bethani I , Michaki V , Krousti K , Rapti G , Efthimiopoulos S . Homer2 and Homer3 interact with amyloid precursor protein and inhibit Aβ production. Neurobiol Dis. 2008;30(3):353‐364. 18387811 10.1016/j.nbd.2008.02.004

[25] Zhang CC , Xing A , Tan MS , Tan L , Yu JT . The role of MAPT in neurodegenerative diseases: genetics, mechanisms and therapy. Mol Neurobiol. 2016;53(7):4893‐4904. 26363795 10.1007/s12035-015-9415-8

[26] Xiao M , Li J , Liu Q , He X , Yang Z , Wang D . Expression and role of TRIM2 in human diseases. Biomed Res Int. 2022;2022:9430509. 36051486 10.1155/2022/9430509 PMC9427271

[27] Deng H , Gao K , Jankovic J . The role of FUS gene variants in neurodegenerative diseases. Nat Rev Neurol. 2014;10(6):337‐348. 24840975 10.1038/nrneurol.2014.78

[28] Bakota L , Brandt R . Tau biology and Tau‐directed therapies for Alzheimer's disease. Drugs. 2016;76(3):301‐313. 26729186 10.1007/s40265-015-0529-0 PMC4757605

[29] Strittmatter WJ , Saunders AM , Goedert M , et al. Isoform‐specific interactions of apolipoprotein E with microtubule‐associated protein tau: implications for Alzheimer disease. Proc Natl Acad Sci U S A. 1994;91(23):11183‐11186. 7972031 10.1073/pnas.91.23.11183 PMC45191

[30] Koutsodendris N , Blumenfeld J , Agrawal A , et al. Neuronal APOE4 removal protects against tau‐mediated gliosis, neurodegeneration and myelin deficits. Nat Aging. 2023;3(3):275‐296. 37118426 10.1038/s43587-023-00368-3 PMC10154214

[31] Nelson MR , Liu P , Agrawal A , et al. The APOE‐R136S mutation protects against APOE4‐driven Tau pathology, neurodegeneration and neuroinflammation. Nat Neurosci. 2023;26(12):2104‐2121. 37957317 10.1038/s41593-023-01480-8 PMC10689245

[32] Holmes BB , Furman JL , Mahan TE , et al. Proteopathic tau seeding predicts tauopathy in vivo. Proc Natl Acad Sci U S A. 2014;111(41):E4376‐E4385. 25261551 10.1073/pnas.1411649111 PMC4205609

[33] Kanno T , Tsuchiya A , Nishizaki T . Hyperphosphorylation of tau at Ser396 occurs in the much earlier stage than appearance of learning and memory disorders in 5XFAD mice. Behav Brain Res. 2014;274:302‐306. 25172181 10.1016/j.bbr.2014.08.034

[34] Nelson MR , Liu P , Agrawal A , et al. The APOE‐R136S mutation protects against APOE4‐driven tau pathology, neurodegeneration and neuroinflammation. Nat Neurosci. 2023;26(12):2104‐2121. 37957317 10.1038/s41593-023-01480-8 PMC10689245

[35] Perez‐Corredor P , Vanderleest TE , Vacano GN , et al. APOE3 Christchurch modulates β‐catenin/Wnt signaling in iPS cell‐derived cerebral organoids from Alzheimer's cases. Front Mol Neurosci. 2024;17:1373568. 38571814 10.3389/fnmol.2024.1373568 PMC10987717

[36] Palomer E , Buechler J , Salinas PC . Wnt signaling deregulation in the aging and Alzheimer's brain. Front Cell Neurosci. 2019;13:227. 31191253 10.3389/fncel.2019.00227 PMC6538920

[37] Mahley RW . Central nervous system lipoproteins: apoE and regulation of cholesterol metabolism. Arterioscler Thromb Vasc Biol. 2016;36(7):1305‐1315. 27174096 10.1161/ATVBAHA.116.307023 PMC4942259

[38] Mahley RW , Ji ZS . Remnant lipoprotein metabolism: key pathways involving cell‐surface heparan sulfate proteoglycans and apolipoprotein E. J Lipid Res. 1999;40(1):1‐16. 9869645

[39] Chen J , Li Q , Wang J . Topology of human apolipoprotein E3 uniquely regulates its diverse biological functions. Proc Natl Acad Sci U S A. 2011;108(36):14813‐14818. 21873229 10.1073/pnas.1106420108 PMC3169138

[40] Liu CC , Zhao N , Yamaguchi Y , et al. Neuronal heparan sulfates promote amyloid pathology by modulating brain amyloid‐b clearance and aggregation in Alzheimer's disease. Sci Transl Med. 2016;8(332):332ra44. 10.1126/scitranslmed.aad3650 PMC5512541 27030596

[41] Bu G . APOE targeting strategy in Alzheimer's disease: lessons learned from protective variants. Mol Neurodegener. 2022;17(1):51. 35922805 10.1186/s13024-022-00556-6 PMC9351235

[42] Zhao N , Liu CC , Qiao W , Bu G . Apolipoprotein E, receptors, and modulation of Alzheimer's disease. Biol Psychiatry. 2018;83(4):347‐357. 28434655 10.1016/j.biopsych.2017.03.003 PMC5599322

[43] Moon HJ , Luo Y , Chugh D , Zhao L . Human apolipoprotein E glycosylation and sialylation: from structure to function. Front Mol Neurosci. 2024;17:1399965. 39169951 10.3389/fnmol.2024.1399965 PMC11335735

[44] Nudelman KNH , Jackson T , Rumbaugh M , et al. Pathogenic variants in the longitudinal early‐onset Alzheimer's disease study cohort. Alzheimers Dement. 2023;19(9):S64‐S73. 37801072 10.1002/alz.13482 PMC10783439

[45] Barbier P , Zejneli O , Martinho M , et al. Role of tau as a microtubule‐associated protein: structural and functional aspects. Front Aging Neurosci. 2019;11:204. 31447664 10.3389/fnagi.2019.00204 PMC6692637

[46] Bergamaschini L , Rossi E , Vergani C , De Simoni MG . Alzheimer's disease: another target for heparin therapy. ScientificWorldJournal. 2009;9:891‐908. 19734963 10.1100/tsw.2009.100 PMC5823143

[47] Chen G , Wang M , Zhang Z , et al. ApoE3 R136S binds to tau and blocks its propagation, suppressing neurodegeneration in mice with Alzheimer's disease. Neuron. 2025;113(5):719‐736.e5. 39814008 10.1016/j.neuron.2024.12.015 PMC12376192

[48] Inestrosa NC , Tapia‐Rojas C , Lindsay CB , Zolezzi JM . Wnt signaling pathway dysregulation in the aging brain: lessons from the octodon degus. Front Cell Dev Biol. 2020;8:734. 32850846 10.3389/fcell.2020.00734 PMC7419590

[49] Caricasole A , Copani A , Caraci F , et al. Induction of dickkopf‐1, a negative modulator of the Wnt pathway, is associated with neuronal degeneration in Alzheimer's brain. J Neurosci. 2004;24(26):6021‐6027. 15229249 10.1523/JNEUROSCI.1381-04.2004 PMC6729239

[50] Ren C , Gu X , Li H , et al. The role of DKK1 in Alzheimer's disease: a potential intervention point of brain damage prevention? Pharmacol Res. 2019;144:331‐335. 31042564 10.1016/j.phrs.2019.04.033

[51] Bao J , Zheng JJ , Wu D . The structural basis of DKK‐mediated inhibition of Wnt/LRP signaling. Sci Signal. 2012;5(224):pe22. 22589387 10.1126/scisignal.2003028 PMC3465688

[52] He KY , Khramtsova EA , Cabrera‐Socorro A , et al. Characterization of APOE Christchurch carriers in 455,306 UK Biobank participants. Mol Neurodegener. 2023;18:92. 38017580 10.1186/s13024-023-00684-7 PMC10685495

[53] Sepulveda‐Falla D , Sanchez JS , Almeida MC , et al. Distinct tau neuropathology and cellular profiles of an APOE3 Christchurch homozygote protected against autosomal dominant Alzheimer's dementia. Acta Neuropathol. 2022;144(3):589‐601. 35838824 10.1007/s00401-022-02467-8 PMC9381462

[54] Chen Y , Song S , Parhizkar S , et al. APOE3ch alters microglial response and suppresses abeta‐induced tau seeding and spread. Cell. 2024;187(2):428‐445 e20. 38086389 10.1016/j.cell.2023.11.029 PMC10842861

[55] Minta K , Brinkmalm G , Janelidze S , et al. Quantification of total apolipoprotein E and its isoforms in cerebrospinal fluid from patients with neurodegenerative diseases. Alzheimers Res Ther. 2020;12(1):19. 32054532 10.1186/s13195-020-00585-7 PMC7020540

[56] Cruchaga C , Kauwe JS , Nowotny P , et al. Cerebrospinal fluid APOE levels: an endophenotype for genetic studies for Alzheimer's disease. Hum Mol Genet. 2012;21(20):4558‐4571. 22821396 10.1093/hmg/dds296 PMC3459471

[57] Cowan CM , Mudher A . Are tau aggregates toxic or protective in tauopathies? Front Neurol. 2013;4:114. 23964266 10.3389/fneur.2013.00114 PMC3741634

