# Supplementary material for: ApoE3 Christchurch and tau interaction as a protective mechanism against Alzheimer's disease
Source: Alzheimers Dement. 2025 Jul 10;21(7):e70396. doi: 10.1002/alz.70396 (PMC12242688; doi:10.1002/alz.70396)
Supplement: Supplementary file 1 — Supporting Information [file ALZ-21-e70396-s002.docx]

Supplemental material for

**ApoE3 Christchurch and tau interaction as a protective mechanism against Alzheimer’s disease**

Paula Perez-corredor*^1^, Said Arevalo-Alquichire*^1^, Randall C. Mazzarino ^1^, Michael O’Hare ^1,2^, Andres F. Muriel, Guido N. Vacano ^3^, Timothy E. Vanderleest ^1^, William P. Miller ^1^, Lina Pineda-Lopez ^1^, Shivani Patel ^1^, Robert A. Obar ^4^, Nihat Polat ^1^, Leo A. Kim ^1^, Joseph F. Arboleda-Velasquez ^+1^; Claudia Marino ^+1,5,6^

This file includes:

**Table S1-S6**

**Figure S1-S6**

**Supplemental methods**

**Table S1**. List of genes, related proteins and downstream pathways differentially linked to *E. coli*-derived ApoE variants

| Gene Name | Protein Name | Biochemical pathway |
| --- | --- | --- |
| NT5DC3 | 5'-Nucleotidase Domain Containing 3 | 5'-nucleotidase activity for dephosphorylation processes (19) |
| BSN | Bassoon | transcriptional regulation, organization and maintenance of the presynaptic release apparatus; Tau propagation (20, 21) |
| PDHB | Pyruvate Dehydrogenase E1 Component Subunit Beta | ATP production (22) |
| HSPD1 | Heat Shock 60kDa Protein 1 | Chaperone activity (23) |
| HOMER2 | Homer Protein Homolog 2 | Receptor trafficking at the synapses (24) |

**Table S2**. List of genes, related proteins and downstream pathways differentially linked to HEK-derived ApoE variants

| Gene Name | Protein Name | Biochemical pathway |
| --- | --- | --- |
| TRIM2 | Tripartite motif-containing protein 2 | Neuroprotection |
| CYCS | Cytochrome C | Mitochondria function/apoptosis |
| GAP43 | Growth associated protein 43 | Neuronal growth, neurogeneration |
| PDXK | Pyridoxal kinase | Vitamin B6 phosphorylation |
| PDIA3 | Protein disuldife-isomerase A3 | Protein folding/ immune response |
| EZR | Ezrin | Surface adhesion |
| KHSRP | KH-type splicing regulatory protein | Regulation of mRNA and microRNA biogenesis |
| CRKL | Crk-like protein | Signaling pathways |
| PCP4 | Purkinje cell protein 4 | Neuronal function |
| CALB1 | Calbindin 1 | Neuronal function |
| ARHGDIA | Rho GDP-dissociation inhibitor alpha | GTPase regulation |
| SH3BGRL3 | SH3 domain-binding glutamic acid-rich-like protein 3 | Cellular processes |
| GABARAPL2 | GABA A receptor-associated protein like-2 | Autophagy |
| MDH1 | Malate dehydrogenase 1 | Citric acid cycle |

**Table S3:** Mass Spectrometry analysis of the MAPT peptides found in the IP fraction of *E.Coli-*derived ApoE3

| **ApoE3 E. Coli-derived** | **n1** | | | **n2** | | **n3** | |
| --- | --- | --- | --- | --- | --- | --- | --- |
| **Peptides** | **ApoE3 WT** | **ApoE3Ch** | **ApoE3 WT** | | **ApoE3Ch** | **ApoE3 WT** | **ApoE3Ch** |
| SGYSSPGSPGTPGSR | 0 | 2 | 3 | | 1 | 2 | 1 |
| SRTPSLPTPPTREPK | 1 | 1 | 1 | | 1 | 2 | 2 |
| TPSLPTPPTR | 0 | 0 | 0 | | 0 | 0 | 1 |
| TPSLPTPPTREPK | 1 | 3 | 1 | | 3 | 1 | 1 |
| LQTAPVPMPDLK | 3 | 3 | 3 | | 2 | 3 | 2 |
| IGSTENLKHQPGGGK | 1 | 1 | 0 | | 0 | 1 | 1 |
| KLDLSNVQSK | 1 | 1 | 2 | | 1 | 1 | 1 |
| LDLSNVQSK | 1 | 0 | 0 | | 0 | 1 | 1 |
| HVPGGGSVQIVYKPVDLSK | 1 | 1 | 0 | | 0 | 1 | 1 |
| SEKLDFKDR | 1 | 1 | 0 | | 0 | 1 | 1 |
| SKIGSTENLK | 0 | 0 | 0 | | 0 | 0 | 1 |
| SKIGSTENLKHQPGGGK | 0 | 0 | 1 | | 1 | 2 | 0 |
| IGSLDNITHVPGGGNK | 2 | 2 | 1 | | 1 | 2 | 1 |
| AKTDHGAEIVYK | 1 | 1 | 1 | | 1 | 1 | 1 |
| TDHGAEIVYK | 1 | 1 | 0 | | 2 | 1 | 1 |
| SPVVSGDTSPR | 0 | 0 | 0 | | 1 | 0 | 1 |
| PVVSGDTSPR | 1 | 0 | 1 | | 0 | 0 | 0 |
| **TOTAL** | **15** | **17** | **14** | | **14** | **19** | **17** |

**Table S4:** Mass Spectrometry analysis of the MAPT peptides found in the IP fraction of HEK*-*derived ApoE3

| **ApoE3 E. HEK-derived** | **n1** | | **n2** | | **n3** | |
| --- | --- | --- | --- | --- | --- | --- |
| **Peptides** | **ApoE3 WT** | **ApoE3Ch** | **ApoE3 WT** | **ApoE3Ch** | **ApoE3 WT** | **ApoE3Ch** |
| [QEFDTMEDHAGDYTLLQDQEGDMDHGLK](https://tmsf.med.harvard.edu/core/www/modules/consensus/?value=sp%7CP10637%7CTAU_MOUSE&pvkey=&search_id=91087) | **0** | **2** | 1 | 1 | 1 | 1 |
| [ESPPQPPADDGAEEPGSETSDAK](https://tmsf.med.harvard.edu/core/www/modules/consensus/?value=sp%7CP10637%7CTAU_MOUSE&pvkey=&search_id=91087) | **0** | **1** | 0 | 0 | 1 | 1 |
| [STPTAEDVTAPLVDER](https://tmsf.med.harvard.edu/core/www/modules/consensus/?value=sp%7CP10637%7CTAU_MOUSE&pvkey=&search_id=91085) | **0** | **0** | 0 | 0 | 0 | 0 |
| [TPPGSGEPPK](https://tmsf.med.harvard.edu/core/www/modules/consensus/?value=sp%7CP10637%7CTAU_MOUSE&pvkey=&search_id=91085) | **0** | **0** | 1 | 1 | 0 | 0 |
| [TPPGSGEPPKSGER](https://tmsf.med.harvard.edu/core/www/modules/consensus/?value=sp%7CP10637%7CTAU_MOUSE&pvkey=&search_id=91087) | **0** | **1** | 0 | 0 | 0 | 0 |
| [SGYSSPGSPGTPGSR](https://tmsf.med.harvard.edu/core/www/modules/consensus/?value=sp%7CP10637%7CTAU_MOUSE&pvkey=&search_id=85577) | 1 | **2** | 1 | 1 | 1 | 3 |
| [TPSLPTPPTR](https://tmsf.med.harvard.edu/core/www/modules/consensus/?value=sp%7CP10637%7CTAU_MOUSE&pvkey=&search_id=91088) | 1 | **2** | 1 | 1 | 1 | 2 |
| [TPSLPTPPTREPK](https://tmsf.med.harvard.edu/core/www/modules/consensus/?value=sp%7CP10637%7CTAU_MOUSE&pvkey=&search_id=91087) | 0 | **1** | 0 | 0 | 0 | 0 |
| [LQTAPVPMPDLK](https://tmsf.med.harvard.edu/core/www/modules/consensus/?value=sp%7CP10637%7CTAU_MOUSE&pvkey=&search_id=91087) | 0 | **2** | 1 | 2 | 1 | 1 |
| [IGSTENLK](https://tmsf.med.harvard.edu/core/www/modules/consensus/?value=sp%7CP10637%7CTAU_MOUSE&pvkey=&search_id=91088) | 1 | **1** | 1 | 1 | 1 | 1 |
| [KLDLSNVQSK](https://tmsf.med.harvard.edu/core/www/modules/consensus/?value=sp%7CP10637%7CTAU_MOUSE&pvkey=&search_id=91087) | 0 | **2** | 1 | 2 | 3 | 3 |
| [HVPGGGSVQIVYKPVDLSK](https://tmsf.med.harvard.edu/core/www/modules/consensus/?value=sp%7CP10637%7CTAU_MOUSE&pvkey=&search_id=91088) | 1 | **1** | 1 | 1 | 1 | 1 |
| [IGSLDNITHVPGGGNK](https://tmsf.med.harvard.edu/core/www/modules/consensus/?value=sp%7CP10637%7CTAU_MOUSE&pvkey=&search_id=91088) | 1 | **1** | 1 | 1 | 0 | 2 |
| [TDHGAEIVYK](https://tmsf.med.harvard.edu/core/www/modules/consensus/?value=sp%7CP10637%7CTAU_MOUSE&pvkey=&search_id=91088) | 1 | **2** | 1 | 2 | 1 | 1 |
| [SPVVSGDTSPR](https://tmsf.med.harvard.edu/core/www/modules/consensus/?value=sp%7CP10637%7CTAU_MOUSE&pvkey=&search_id=91087) | 0 | **1** | 0 | 0 | 0 | 1 |
| **TOTAL** | **6** | **19** | **10** | **13** | **11** | **17** |

**Table S5.** Target proteins supporting the prediction of the MAPT upstream regulator in the mechanistic network.

| Target | Expr Log Ratio | Molecule Type | MAPT | PSEN2 | APP | PSEN1 | CTNNB1 | TP53 |
| --- | --- | --- | --- | --- | --- | --- | --- | --- |
| AAK1 | 0.777 | kinase | Affected |  |  |  |  |  |
| ACADL | -0.769 | enzyme |  |  |  |  | Inhibited | Activated |
| ACAT1 | 0.154 | enzyme | Affected |  |  |  | Affected | Activated |
| ACO2 | 1.233 | enzyme | Affected |  | Affected | Affected |  | Affected |
| ACTB | -0.315 | other | Affected | Activated | Inhibited | Activated | Affected | Inhibited |
| ACTN1 | -1.88 | transcription regulator | Affected |  |  |  |  | Affected |
| ADD2 | -0.794 | other | Affected |  |  |  |  |  |
| ALDOA | 3.066 | enzyme | Affected |  | Affected | Affected |  | Inhibited |
| AMPH | -2.555 | other | Affected |  | Affected | Affected |  |  |
| APOE | -1.733 | transporter |  |  | Inhibited | Activated |  | Activated |
| APP | 1.496 | other | Affected | Activated | Activated | Activated | Affected | Activated |
| ATP5F1A | -0.25 | transporter | Affected |  | Affected | Affected |  | Activated |
| ATP5F1B | -0.131 | transporter | Affected |  | Affected | Affected |  |  |
| ATP5F1C | 0.371 | transporter | Affected |  | Affected | Affected |  | Activated |
| ATP5F1D | 0.563 | transporter | Affected |  | Affected | Affected |  |  |
| ATP6V1A | -0.338 | transporter | Affected |  | Affected | Affected |  |  |
| ATP6V1E1 | 0.161 | transporter | Affected |  | Affected | Affected |  |  |
| BASP1 | 0.589 | transcription regulator | Affected |  | Affected | Affected |  |  |
| BIN1 | 0.348 | other |  |  | Affected |  |  |  |
| Calm1 (includes others) | -0.874 | other |  |  |  |  |  | Affected |
| CARM1 | -0.818 | transcription regulator | Affected |  | Affected | Affected |  |  |
| CFL1 | 0.15 | other | Affected |  | Affected | Affected |  |  |
| CFL2 | 0.085 | other | Affected |  | Affected | Affected |  |  |
| CKB | 2.895 | kinase | Affected |  | Affected | Affected |  | Activated |
| CLTC | 1.158 | other | Affected |  | Affected | Affected |  | Affected |
| CORO1A | 0.855 | other | Affected |  |  |  |  | Activated |
| CPLX1 | -0.362 | transporter |  |  | Affected |  |  |  |
| CRYM | 1.174 | enzyme | Affected |  | Affected | Affected |  |  |
| CTTN | 1.051 | other |  |  | Affected |  |  |  |
| DBN1 | 2.812 | other |  |  | Inhibited |  |  |  |
| DLST | -1 | enzyme |  |  |  |  |  | Activated |
| DMXL2 | 0.03 | other | Affected |  |  |  |  |  |
| DNM1 | -1.134 | enzyme |  |  | Affected |  |  | Inhibited |
| DNM1L | -4.282 | enzyme | Affected |  | Activated | Affected |  | Activated |
| DPYSL2 | -1.035 | enzyme | Affected |  | Inhibited | Affected |  |  |
| DYNC1LI1 | 1.348 | other | Affected |  |  |  |  |  |
| EEF1A1 | 0.162 | translation regulator |  |  |  |  |  | Activated |
| ENO1 | 2.074 | enzyme | Affected |  | Affected | Affected |  |  |
| ENO2 | 1.292 | enzyme | Affected |  | Affected | Affected | Activated | Affected |
| ETFA | -1.373 | transporter |  |  |  |  |  | Inhibited |
| Ewsr1 | 1.24 | other |  |  |  |  | Affected |  |
| FUBP1 | 0.047 | transcription regulator |  |  |  |  |  | Inhibited |
| GAPDH | 1.509 | enzyme | Affected | Inhibited | Affected | Inhibited | Inhibited | Activated |
| GLUL | 0.059 | enzyme |  |  |  |  | Activated | Activated |
| GOT2 | -3 | enzyme | Affected |  | Affected | Affected |  |  |
| GPHN | 1.332 | enzyme |  |  |  |  |  | Affected |
| Hbb-b1 | -0.533 | transporter | Affected | Affected | Affected | Affected |  |  |
| Hbb-b2 | 1.228 | enzyme | Affected |  | Affected | Affected |  |  |
| HINT1 | 0.691 | enzyme | Affected |  | Affected | Affected |  |  |
| HSP90AA1 | -1.693 | enzyme | Affected |  | Affected | Affected |  | Activated |
| HSP90AB1 | -0.906 | enzyme |  |  | Affected |  |  | Activated |
| HSPA5 | -3.604 | enzyme | Affected |  | Inhibited | Affected |  | Activated |
| HSPA8 | -1.074 | enzyme | Affected |  | Affected | Affected |  | Activated |
| HSPE1 | 0.909 | enzyme | Affected |  | Affected | Affected | Affected |  |
| LDHB | -2.673 | enzyme | Affected |  | Affected |  |  | Activated |
| MAP1A | -7.703 | other | Activated |  |  |  |  |  |
| MAP1B | -4.447 | other | Activated |  | Inhibited |  |  |  |
| MAP2 | -0.928 | other | Activated |  | Activated | Affected |  |  |
| MAP6 | -0.443 | other | Affected |  | Affected | Affected |  |  |
| MAPK1 | -0.242 | kinase | Affected |  | Affected | Affected |  | Inhibited |
| MAPK3 | 0.279 | kinase | Activated |  |  |  |  | Affected |
| MAPRE3 | -0.069 | enzyme |  |  |  |  |  | Inhibited |
| MAPT | -0.696 | other | Inhibited |  | Inhibited | Affected | Affected | Inhibited |
| MBP | -1.363 | other | Affected | Affected | Affected | Affected |  |  |
| MDH2 | 3.582 | enzyme |  |  |  |  |  | Affected |
| ME2 | 1.036 | enzyme |  |  |  |  |  | Inhibited |
| MECP2 | -0.985 | transcription regulator |  |  | Affected |  |  |  |
| MRPL12 | 0.51 | other |  |  |  |  |  | Inhibited |
| MYH10 | 2.034 | enzyme |  |  |  |  |  | Inhibited |
| NME1 | -0.199 | kinase | Affected |  | Affected | Affected |  | Inhibited |
| Nrgn | -0.051 | other |  |  | Affected |  |  |  |
| OGDH | 0.506 | enzyme | Affected |  |  |  |  | Inhibited |
| PACSIN1 | -1.379 | kinase |  |  | Affected |  |  | Affected |
| PARK7 | -1.176 | enzyme | Affected |  |  |  |  | Affected |
| PCP4 | 0.631 | other | Affected |  | Affected | Affected |  |  |
| PFKM | 2.965 | kinase |  |  |  |  |  | Inhibited |
| PFKP | 1.203 | kinase |  |  | Activated |  |  | Inhibited |
| PFN2 | 0.513 | enzyme | Affected |  |  |  |  |  |
| PPP1R1B | 0.466 | phosphatase | Affected |  |  |  |  |  |
| PPP3CA | -0.83 | phosphatase |  |  |  |  | Inhibited | Activated |
| PPP3R1 | -2.492 | phosphatase | Inhibited |  |  |  |  |  |
| PURA | -7.03 | transcription regulator |  |  | Affected |  |  | Activated |
| RAB3A | 0.065 | enzyme |  |  |  |  | Affected |  |
| RPL18 | -5.376 | other |  |  |  |  | Affected |  |
| RPLP2 | 0.256 | other |  |  |  |  | Affected |  |
| RPS10 | 1.931 | other |  |  |  |  | Affected |  |
| RPS11 | -2.029 | other |  |  |  |  | Affected |  |
| RPS19 | 0.689 | other |  |  |  |  | Affected | Activated |
| RPS3 | 0.356 | enzyme |  |  |  |  | Affected | Activated |
| SDHA | 1.237 | enzyme |  |  |  |  | Inhibited | Inhibited |
| SF3A1 | 1.755 | other |  |  |  |  | Affected |  |
| SFPQ | 0.504 | other |  |  |  |  |  | Affected |
| SH3GL2 | -1.245 | enzyme | Affected |  | Affected | Affected |  |  |
| SNAP25 | -0.118 | transporter | Affected |  | Affected | Affected |  |  |
| SNAP91 | -1.309 | other | Affected |  |  |  |  |  |
| SNCA | -0.208 | enzyme | Affected |  | Affected | Activated |  | Inhibited |
| SNCB | 0.189 | other | Affected |  | Affected | Inhibited |  |  |
| SPTAN1 | -1.109 | other | Affected |  | Affected | Affected |  |  |
| ST13 | -1.046 | other |  |  |  |  |  | Inhibited |
| STX1B | 0.946 | other | Affected |  | Affected | Affected |  |  |
| STXBP1 | -1.046 | transporter | Affected |  | Affected | Affected | Affected |  |
| SUCLA2 | 0.846 | enzyme |  |  |  |  |  | Inhibited |
| SUCLG1 | 1.453 | enzyme | Affected |  | Affected | Affected |  | Activated |
| SYN1 | 0.459 | other | Affected |  | Inhibited | Affected |  | Activated |
| SYN2 | 0.47 | other |  |  | Affected |  |  |  |
| SYNJ1 | -0.478 | phosphatase | Affected |  |  |  |  |  |
| TIMM9 | 1.227 | transporter |  |  |  |  |  | Inhibited |
| TPI1 | 1.541 | enzyme | Affected |  | Affected | Affected |  |  |
| Tpm1 | -0.018 | other | Affected |  | Affected | Affected |  | Inhibited |
| Tpm2 | -1.132 | other | Affected |  | Affected | Affected |  | Inhibited |
| TPM3 | 1.105 | other | Affected |  | Affected | Affected |  | Activated |
| TPPP | 0.009 | enzyme | Affected |  | Affected | Affected |  |  |
| TSC22D1 | 1.944 | transcription regulator |  |  |  |  | Activated |  |
| TUBA1A | -3.3 | other | Affected |  | Affected | Affected |  |  |
| TUBB | -1.811 | other | Affected |  | Affected | Affected |  | Inhibited |
| TUBB2A | -2.177 | other | Affected |  | Affected | Affected |  |  |
| TUBB2B | -2.442 | other | Affected |  | Affected | Affected | Affected |  |
| TUBB3 | -3.956 | other | Affected |  | Inhibited | Affected |  | Affected |
| TUBB4A | -2.315 | other |  |  |  |  |  | Affected |
| TUBB4B | -1.968 | other | Affected |  | Affected | Affected |  | Affected |
| UBA1 | -0.778 | enzyme |  |  |  |  | Affected | Activated |
| UCHL1 | 1.05 | peptidase | Affected |  | Affected | Affected |  |  |
| WASF1 | 0 | other | Affected |  | Affected |  |  |  |
| YWHAB | 0.887 | other |  |  | Affected |  |  |  |
| YWHAE | -0.132 | other | Affected |  | Affected | Affected |  |  |
| YWHAG | 1.496 | other |  |  |  |  |  | Affected |
| YWHAH | 0.455 | transcription regulator |  |  |  |  |  | Activated |
| YWHAZ | -0.56 | enzyme | Affected |  | Affected | Affected |  | Affected |

**Table S6:** Upstream regulators of HEK-derived ApoE3Ch obtained from Ingenuity Pathway Analysis (IPA) of HEK-derived ApoE3Ch interactome.

| **Upstream Regulator** | **Molecule Type** | **Activation z-score** | **p-value of overlap** |
| --- | --- | --- | --- |
| MAPT | other | 1.941 | 7.09E-87 |
| APP | other | 0.109 | 1.33E-57 |
| PSEN1 | peptidase | 0.816 | 1.11E-47 |
| LARP1 | translation regulator | 4.116 | 1.11E-41 |
| MLXIPL | transcription regulator | -5.238 | 1.05E-38 |
| FMR1 | translation regulator | 3.434 | 1.86E-37 |
| TP53 | transcription regulator | -0.949 | 1.24E-36 |
| MYC | transcription regulator | -4.779 | 9.62E-32 |
| RICTOR | other | 3.041 | 1.16E-31 |
| LH (complex) | complex | -3.333 | 1.57E-30 |
| NFE2L2 | transcription regulator | -1.965 | 6.32E-26 |
| CLPP | peptidase | 3.4 | 1.22E-23 |
| MMP12 | peptidase |  | 5.39E-20 |
| **CTNNB1** | **transcription regulator** | **-1.485** | **2.65E-19** |
| MTOR | kinase | -0.89 | 5.49E-19 |
| PCGEM1 | other | -2.531 | 8.75E-17 |
| SMYD1 | transcription regulator |  | 1.33E-16 |
| TCR (complex) | complex | -1.897 | 1.87E-15 |
| RTN4 | other | -0.839 | 1.22E-14 |

Figure S1. Optimization of immunoprecipitation conditions for pull-down of mouse brain proteins with ApoE3 variants. **A.** Experimental design of the mouse brain co-immunoprecipitation using three different lysis buffers (n=1). **B**. Coomassie staining of the immunoprecipitated and unbound proteins. Red boxes indicate bands that were excised for mass spectrometry analysis. **C.** Number of ApoE3WT and ApoE3Ch interactor proteins in each lysis buffer. **D.** Buffer 3 PAGE and western blot: Coomassie native gel, Silver stain native gel, Silver stain denatured reducing gel, native Western blots: anti His-tag and anti ApoE. IP: Immunoprecipitation, UB: Unbound.

Figure S2. Mass spectrometry fragment analysis of the interaction between MAPT and ApoE. **A.** Amino acid sequence of MAPT. **B.** Representative alignment of MAPT fragments detected via mass spectrometry with the mouse database. **C.** Summary table of the predicted fragment pattern for LQTAPVPM*PDLK. **D.** Representative scatter plot showing the data distribution expressed as part per million (PPM) over mass (m/z). **E**. Representative chromatogram of the detected fragments via mass spectrometry.

Figure S3. **A.** Anti Tau-5 ELISA of binding profiles of logarithmic concentrations of pre-formed protofibrils of tau interacting to either ApoE3 WT (black curve) or ApoE3Ch (grey curve). **B.** Area under the curve (AUC) analysis of the ELISA binding curves presented in panel A confirmed the significant increased binding of tau to ApoE3Ch as compared to ApoE3 WT (p < 0.0001, One-way ANOVA followed by Fisher’s LSD test for multiple comparisons). **C, D.** Sensorgram curves for protein binding of ApoE3WT (C) and ApoE3Ch (D) and pre-formed tau fibrils obtained via BLI-kinetic assay.

Figure S4. ApoE3 variants modulate tau phosphorylation of tau biosensor cells. **A**. Representative western blot of pTau (S396) from Tau RD P301S biosensor cells. Arrows point the studied bands. **B-D**. Phosphorylation levels of tau aggregates of (B) 250 kDa, (C) 110 kDa, (D) 54 kDa. (*p≤0.05, **p≤0.01, ***p≤0.001, ****p≤0.0001, one-way ANOVA, Fisher LDS post-hoc test).


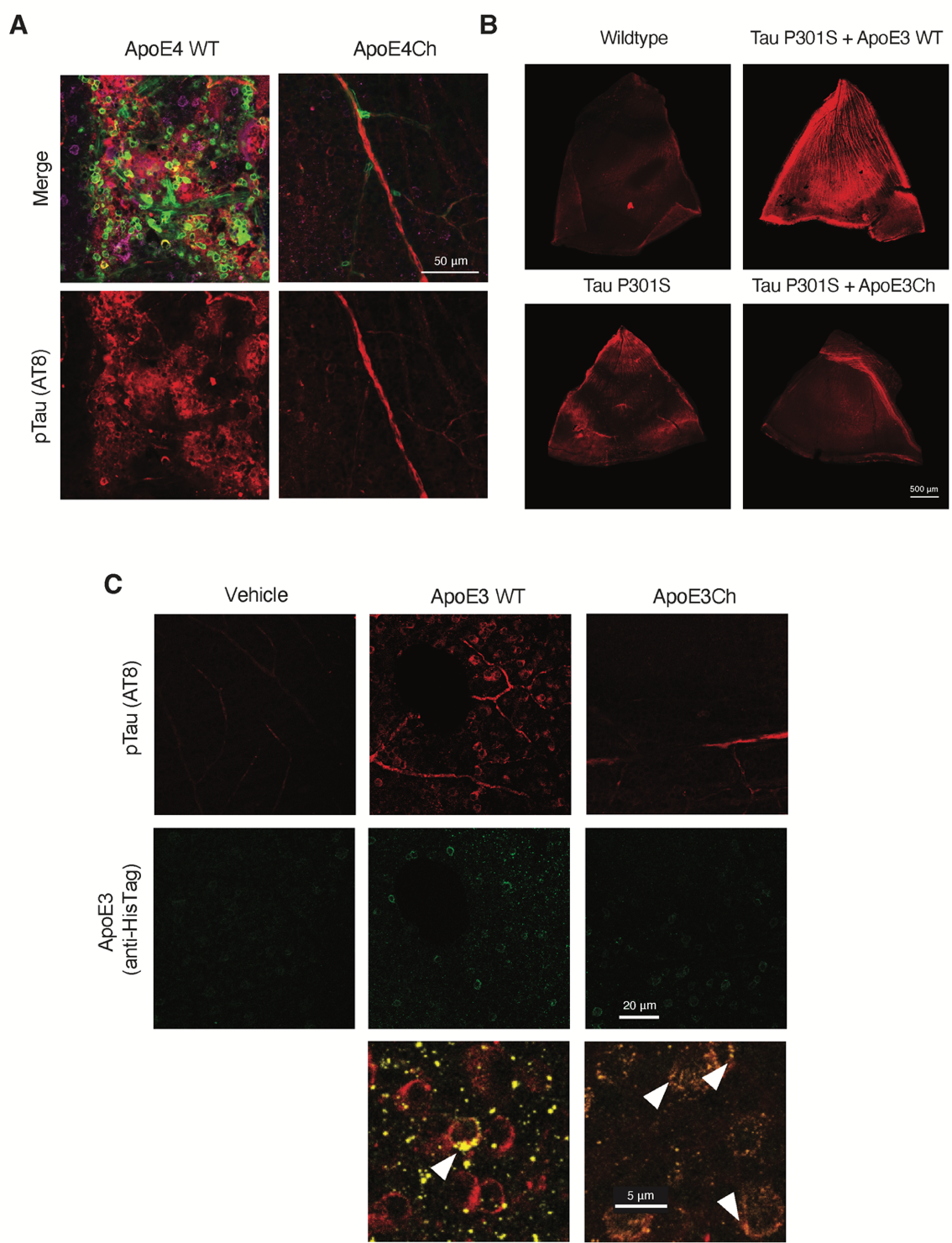


Figure S5. **A.** Representative immunofluorescence images of tau phosphorylation (anti-AT8) of 40-days old P301S mice treated with ApoE4 variants. Scale bar= 50 μm. **B.** Representative immunofluorescence images of tau phosphorylation (S396) of 40 days old mice treated with the ApoE3 variants. Scale bar= 500 μm. **C**. Representative images of pTau (AT8) and recombinant ApoE3 variants (detected using an anti-His-Tag antibody) showed colocalization of both signals in the mouse retinas (white arrows). Scale bars= 20 and 5 μm.

Figure S6. Validation of the interaction between ApoE and Dkk1. **A.** Representative western blotting of co-immunoprecipitation (Ni-IP) of recombinant ApoE3Ch and ApoE3 WT to human Dkk1. Positive bands were detected using anti-ApoE (top blot) and anti-Dkk1 (bottom blot) antibodies. As control, inputs were also analyzed. **B, C**. Densitometry measurements of immunoprecipitated fractions normalized to input detected using (B) anti ApoE and (C) anti Dkk1 antibody. Data expressed as normalized to input fractions and statistical differences are quantified via unpaired Student’s T Test (*p=0.0362). **D-F**. Isothermal titration calorimetry (ITC) analysis of titrated concentrations of ApoE3WT variants in the presence of a constant concentration of Dkk1 using lower (D) and higher (F) range of concentrations. Data is expressed as corrected heat rate (mJ/s) over time (s). Negative values are indicative of exothermic interactions. **E- G**. Integrated hits of the ITC data presented in panels (D) and (F), expressing the interaction profile between the two proteins as kJ/mole of Dkk1 over the mole ratio of Dkk1 over ApoE3WT. **H**. Wnt signaling reporter HEK293 cell line was tested against recombinant huApoE3WT or huApoE3Ch with huWnt3a and huDkk1. Wells were tested in triplicate for each individual experiment over three independent experiments.

**Supplemental Methods**

**Bio-layer interferometry (BLI)**

ApoE3 proteins were immobilized on HISIK Biosensors (Pall ForteBio), followed by a washing step with loading buffer (1Å~PBS, pH7.4, with 0.1% BSA and 0.02% Tween-20). Six concentrations of analytes, 62.5 nM, 125 nM, 250 nM, 500 nM, 1,000 nM, and 2,000 nM were loaded into BLI system, and the assay was performed in solid black 96-well plates (Greiner Bio-One), using agitation set at 1,000 rpm. The steps of the assay were the following: baseline 1 (buffer), loading (ligand), baseline 2 (buffer), association (analyte), and dissociation (buffer). The experimental data were fit with the 1:1 binding model and analyzed with global fitting using Octet Data Analysis software to calculate KD.

**Wnt reporter assay**

Wnt signaling reporter assay was performed according to manufacturer recommended protocol and as previously described (29). HEK293 TCF/LEF luciferase Wnt Signaling reporter cell line was commercially sourced (BPS Bioscience, cat. 60501) along with growth (BPS Bioscience, cat. 79531) and assay (BPS Bioscience, cat. 60187) media as well as Wnt3a (R&D Systems, cat. 5036-WN-010). Wnt3a was aseptically reconstituted in sterile filtered 1x dPBS with 0.1%BSA then aliquots were frozen at -80°C. Human recombinant ApoE3-His protein constructs and reelin-Fc recombinant constructs were sourced as fee for service from Innovagen AB (Lund, Sweden). Reporter assay was performed using three replicates per condition per experiment and repeated over six separate experiments. Luciferase Assay System (Promega, cat. E1501) was used to detect luciferase production and was measured on Biosystem H1 bioanalyzer.

**Isothermal titration calorimetry (ITC)**

ITC experiments of recombinant Dkk1 and ApoE3 (WT or Christchurch) variants were carried out at 25°C in a buffer containing 20 mM Sodium phosphate (pH 7.5) as a fee-for-service at Ichor Life Sciences, Inc. 150 mM NaCl (ITC buffer) on a nano ITC low volume isothermal calorimeter from TA instruments. ApoE3WT and Dkk1 were buffer exchanged into ITC buffer using PD10 desalting column (Cytiva). The concentrations of ApoEE3 WT and Dkk1 were determined using the BCA assay. The concentration of ApoE3 WT in the ITC cell was either 10 µM or 29 µM. Dkk1 was titrated using a concentration of either 50 µM or 151 µM. The titration schedule consisted of one at 0.25 μL and 20 at 2.5 μL volume of injections into the ITC cell with 300 s intervals between injections. Enthalpy changes after each injection were calculated using the NanoAnalyze software (TA instruments).
